# Predicting blooms of toxic cyanobacteria in eutrophic lakes with diverse cyanobacterial communities

**DOI:** 10.1038/s41598-017-08701-8

**Published:** 2017-08-21

**Authors:** Aleksandra Bukowska, Tomasz Kaliński, Michał Koper, Iwona Kostrzewska-Szlakowska, Jan Kwiatowski, Hanna Mazur-Marzec, Iwona Jasser

**Affiliations:** 1 Department of Microbial Ecology & Environmental Biotechnology, Faculty of Biology, Biological and Chemical Research Centre, University of Warsaw, Żwirki i Wigury 101, 02-089 Warszawa, Poland; 2 Institute of Genetics and Biotechnology, Faculty of Biology, University of Warsaw, A. Pawińskiego 5a, 02-106 Warszawa, Poland; 3 Faculty of Biology, University of Warsaw, I. Miecznikowa 1, 02-096 Warszawa, Poland; 4 Department of Phylogenetics and Evolution, Faculty of Biology, Biological and Chemical Research Centre, University of Warsaw, Żwirki i Wigury 101, 02-089 Warszawa, Poland; 5 Department of Marine Biotechnology, Institute of Oceanography, University of Gdańsk, al. Marszałka Piłsudskiego 46, 81-378 Gdynia, Poland; 6 Department of Plant Ecology & Environmental Conservation, Faculty of Biology, Biological and Chemical Research Centre, University of Warsaw, Żwirki i Wigury 101, 02-089 Warszawa, Poland

## Abstract

We investigated possibility of predicting whether blooms, if they occur, would be formed of microcystin-producing cyanobacteria. DGGE analysis of 16S-ITS and *mcy*A genes revealed that only *Planktothrix* and *Microcystis* possessed *mcy*-genes and *Planktothrix* was the main microcystin producer. qPCR analysis revealed that the proportion of cells with *mcy*-genes in *Planktothrix* populations was almost 100%. Microcystin concentration correlated with the number of potentially toxic and total *Planktothrix* cells and the proportion of *Planktothrix* within all cyanobacteria, but not with the proportion of cells with *mcy*-genes in total *Planktothrix*. The share of *Microcystis* cells with *mcy*-genes was low and variable in time. Neither the number of *mcy*-possessing cells, nor the proportion of these cells in total *Microcystis*, correlated with the concentration of microcystins. This suggests that it is possible to predict whether the bloom in the Masurian Lakes will be toxic based on *Planktothrix* occurrence. Two species of toxin producing *Planktothrix*, *P. agardhii* and *P. rubescens*, were identified by phylogenetic analysis of 16S-ITS. Based on morphological and ecological features, the toxic *Planktothrix* was identified as *P. agardhii*. However, the very high proportion of cells with *mcy*-genes suggests *P. rubescens*. Our study reveals the need of universal primers for *mcy*A genes from environment.

## Introduction

Blooms of algae, including cyanobacteria, are one of the consequences of eutrophication^1, 2^. They create various problems, such as decreasing water transparency and species diversity of phytoplankton and other organisms, high values of production and respiration, high oxygen consumption and formation of anaerobic zones, accumulation of toxic hydrogen sulfide at the bottoms of reservoirs and the occurrence of unpleasant odors, and, finally, the presence of various toxins. The latter is particularly detrimental, because toxins produced by algae are dangerous to aquatic organisms and can be hazardous for human health and farm animals, who drink water from the reservoirs. In freshwater ecosystems, cyanobacteria constitute the main group of phytoplankton organisms responsible for toxic blooms^3^. Eutrophication and climate change, which involve the increase of air and water temperature and higher runoff from the catchment, bring about the occurrence of cyanobacterial blooms. The blooms take place more often, last longer and become toxic more often^4, 5^.

**Figure 1:**
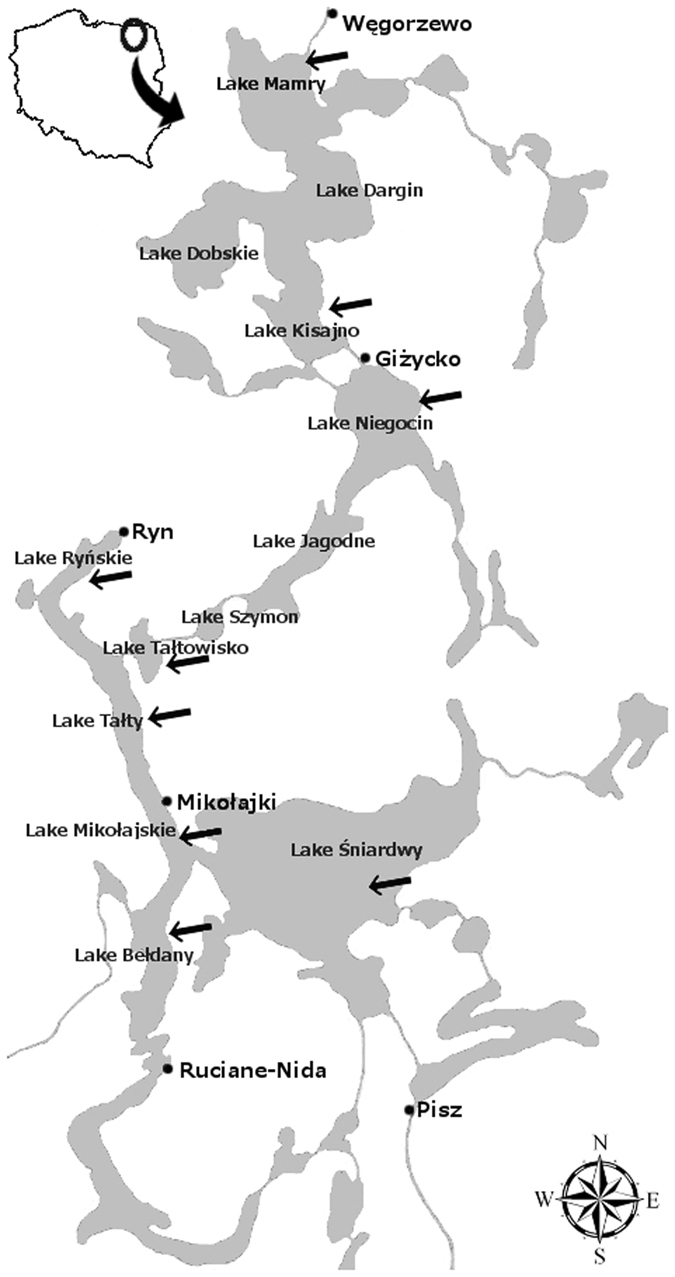
Study area – the Great Masurian Lakes system. Arrows indicate lakes analyzed in this study. Map prepared in GIMP 2.8.16 software https://www.gimp.org/.

The Great Masurian Lake (GML) system in North-Eastern Poland, comprising several large lakes interconnected by canals, are used intensively for recreation (Fig. 1). The lakes are a holiday destination for hundreds of thousands of people and serve as the pillar of the local economy, which relies on tourism. The outflow of the lakes is connected to the Narew River, which is the main tributary of the Vistula River, a source of drinking water for Warsaw, and one of the last unregulated river systems in Europe.

The cyanobacterial communities in the GML are diverse^6–10^ and often dominate phytoplankton. Most of the cyanobacterial species in these lakes are potentially toxic. Therefore, it is important to determine whether there are toxins in lake waters or if there is a risk that the cyanobacteria will produce toxins. The ability of a cyanobacterial community to produce toxins can be identified either by the direct analysis of toxins in the water or by the analysis of the genes involved in the biosynthesis of the toxins^11^. These so-called toxicity genes are responsible directly or indirectly for the production of various toxins (e.g. the *mcy*-gene cluster for microcystins; the *sxt*, *cyr* and *ana* genes for saxitoxin, cylindrospermopsin and anatoxin-a). In the case of microcystins (which are the most frequently-occurring freshwater toxins), genes from the *mcy*-gene cluster are routinely assayed^3^. Eight genes, *mcy*A, B, C, D, E, G, H, J, are always present and are essential for toxin production^11^.

Information about the number of cells with toxicity genes, without information of the share of potentially ‘toxic’ genotypes in a given taxon of cyanobacteria, allow the recognition that the community is presently formed by toxic cyanobacteria, but it is not enough to predict whether the bloom, if it occurs, will be toxic. Verification of the proportion of toxic cells requires precise tools, which make it possible to count potentially toxic cells and the overall number of cells from a particular taxon. Quantitative PCR (qPCR), using taxa-specific probes allows the estimation of both the total number of cells and the cell number from this taxon with a specific genotype ‒ in this case, possessing ‘toxicity genes’ in the genome. According to the hypothesis of Kurmayer *et al*.^12^, the share of genotypes in the environment bearing *mcy* genes is stable during the vegetation season. Therefore, on the basis of the proportion of potentially ‘toxic’ genotypes in spring, it should be possible to predict whether the bloom, if it occurs later during the season, will be toxic.

There are few studies that have investigated relationships between the proportion of *mcy*-genotypes in the population of cyanobacteria and the concentrations of microcystins in waters^13^. Even less is known about the risk of toxic cyanobacterial blooms and the concentrations of toxins in the GML. Mankiewicz *et al*.^14^ showed that Masurian waters are not free of microcystins and that their concentrations varied during one sampling from 4 to 12 µg L^−1^. Recently, genes from the *mcy*-gene cluster were detected in four of the Masurian Lakes during the entire vegetation season^9^.

The main objective of our study was to determine – on the basis of the share of cells with toxicity genes in the total number of given cyanobacteria taxon – whether we can predict if the bloom of cyanobacteria, if it occurs, will be toxic. The second objective of the study was to reveal the relationships between environmental parameters and the occurrence of cyanobacteria bearing toxicity genes, their contribution to the total cyanobacteria community, and microcystin (MC) concentrations in the studied lakes. To obtain our results, we investigated: (1) the biomass, structure and contribution of cyanobacteria in the phytoplankton of studied lakes; (2) which taxa are potentially toxic and are responsible for the toxic bloom; (3) if and when genotypes with toxicity genes from the *mcy*-gene cluster occur in the waters of the Great Masurian Lake system and to which cyanobacterial taxon they belong; (4) the number of cells with toxicity genes in the total number of a given taxon, and how this proportion changes over the season and within the two years of the study; and finally (5) if and what concentrations of microcystins accompany the potentially toxic cyanobacteria in these waters.

## Results

During the study period, the trophic status of the studied lakes varied between advanced mesotrophy and eutrophy, with mean trophic state index (TSI) values between 47 ± 4 and 60 ± 4 (Table S1). The maximal and minimal values of TSI were noted in the spring. During summer, the differences between TSI in the studied lakes were smaller, but the respective lakes could still be classified within the same trophic status category. During the course of the study, the northern lakes – Mamry and Kisajno – represented the lowest trophic status, while lakes Niegocin and Tałtowisko could be characterized as slightly eutrophic; and Lake Śniardwy was characterized as eutrophic, and Mikołajskie, Bełdany and Tałty as highly eutrophic. The chlorophyll *a* concentrations in the studied lakes varied from 6 to over 40 µg L^−1^, and in five of the eight studied lakes, were above 20 µg L^−1^ during most of the vegetation season. In all studied lakes in the GML system, in 58 out of 64 samples, the PCR analyses revealed the presence of the *mcy*A*, mcy*D and *mcy*E genes (Table S2).

### Phytoplankton biomass, composition and structure

Total phytoplankton biomass varied between 0.6 ± 0.5 mg L^−1^ of wet weigh in Lake Niegocin in the spring and 5.7 ± 2.3 mg L^−1^ in Lake Tałty in the summer (Table S1). Bacillariophyceae and occasionally Cryptophyceae dominated among the eukaryotic phytoplankton in the studied lakes in the spring, while Dinophyceae prevailed among the eukaryotes during the summer. *Cyclotella* spp., *Tabellaria flocculosa* var. *asterionelloides, Asterionella formosa* and *Aulacoseira granulata* prevailed in the Bacillariophyceae community. Cryptophyceae, which dominated occasionally in a few of the lakes in the spring, were represented by *Cryptomonas ovata* and *Rhodomonas lacustris*. *Ceratium hirundinella* dominated among Dinophyceae and occasionally in the total phytoplankton biomass.

During the summer, cyanobacteria accounted on average for 41% ± 12 of the total phytoplankton biomass (Table S1), but the maximal share was as much as 82–91% of the total phytoplankton biomass (91% in Lake Mamry in August 2011; 83% in Lake Kisajno in July 2012; 82% in Lake Tałtowisko in August 2012). Microscopic analysis made the identification of 44 species of cyanobacteria possible. Among the cyanobacterial order Oscillatoriales, *Pseudanabaena limnetica* was the dominating species, prevailing in the biomass in most of the lakes. *Planktolyngbya limnetica*, *Limnothrix redekei* and *Planktothrix agardhii* and *P. agardhii* (var.) *suspensa* were also occasionally plentiful, constituting up to 50% of all Oscillatoriales. In less eutrophicated lakes in the spring and summer, and in the spring in more eutrophicated lakes, Chroococcales and Synechococcales contributed substantially to the cyanobacterial population. Among the Synechococcales, picocyanobacteria, represented by *Synechococcus* and *Cyanobium*, dominated in quantity and in the biomass, contributing between 60 and 95% to this group. From among the Chroococcales, *Snowella lacustris*, *S. litoralis* and *Aphanocapsa* spp. were noted in most of the lakes, mostly in July, while *Microcystis aeruginosa* was present in the northern, less eutrophicated lakes, and only occasionally occurred in the more eutrophic ones in the middle and southern part of the GML system. *M. smithii*, *M. wesenbergii* and *M. ichtyoblabe* were also noted on a few occasions.

From the 44 species of cyanobacteria identified in the samples, 16 were potentially microcystin-producing (including two species of *Planktothrix*: *agardhii* and *agardhii* (var.) *suspensa*, six species of *Microcystis*, *Pseudanabaena limnetica, Snowella lacustris*, *Aphanocapsa* spp., *Dolichospermum flos-aquae* and *Limnothrix redekei*). Six species were potential producers of neurotoxins or other unknown toxins (Table S3).

### Phylogenetic analysis of DGGE-derived ITS and *mcy*A sequences – diversity of toxicity gene-bearing cyanobacteria vs. other cyanobacteria

**Figure 2:**
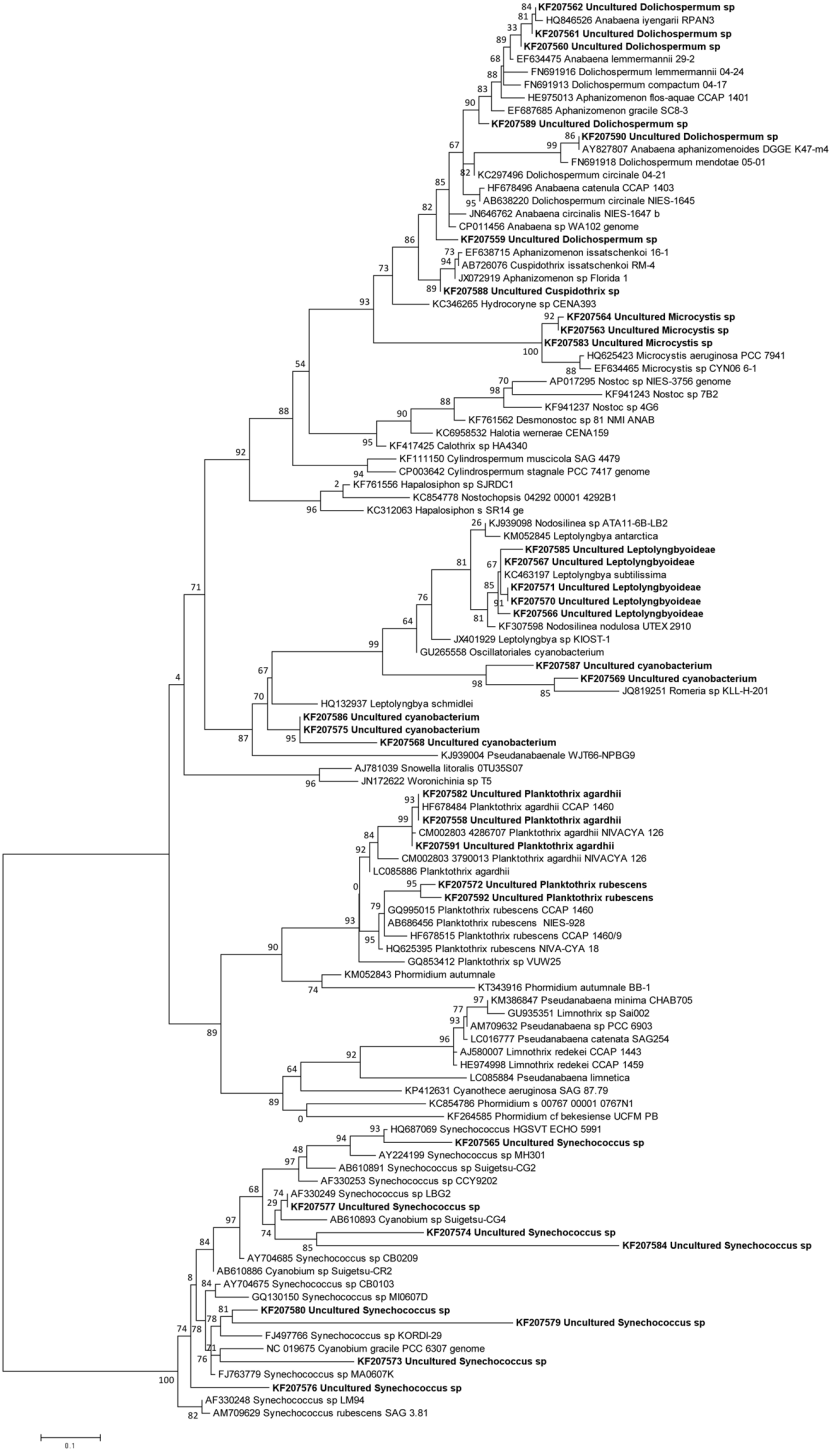
Phylogenetic tree obtained with partial 16S and ITS rRNA sequences corresponding to the sequences obtained from DGGE bands. Numbers at nodes represent branch frequencies. Evolutionary trees, involving 110 sequences, were estimated by the PhyML program^49^, using the Maximum Likelihood method based on the General Time Reversible model with a discrete Gamma distribution model for evolutionary rate differences among sites (4 categories (+G, Gamma shape parameter = 0.730)). Branch frequencies were calculated by aLRT test, according to Anisimova and Gascuel^50^. The tree with the highest log likelihood (−6266.296) is drawn to scale, with branch lengths measured in the number of substitutions per site. The rate variation model allowed for some sites to be evolutionarily invariable (+I, Proportion of invariant sites = 0.071).

Phylogenetic analysis of ITS sequences obtained from DGGE bands revealed that most of the OTUs could be assigned to known cyanobacterial genera and/or species. The tree (Fig. 2) shows that eight of the OTUs belong to the *Cyanobium*/*Synechococcus* clade and seven to the *Dolichospermum*/*Aphanizomenon/Cuspidothrix* clade. Within this clade, one sequence grouped with *Cuspidothrix issatschenkoi* sequences with high probability. Since *C. issatschenkoi* was detected by morphological analyses, it is possible that this sequence might come from this species. We were able to assign eight sequences to Leptolyngbyoideae and two others clustered with *Romeria* sp., though the phylogenetic position of *Romeria* was questionable on our tree. Five other sequences were assigned to *Planktothrix* and three to *Microcystis*. Therefore, all major groups of cyanobacteria identified microscopically were represented in the ITS-DGGE phylogenetic tree. However, neither *Planktolyngbya* nor *Pseudanabaena* were identified by phylogenetic analysis. This might be due to the so far unresolved phylogenetic questions concerning most cyanobacterial genera, including *Leptolyngbya*, *Planktolyngbya* and *Pseudanabaena* ^15^. Therefore, the sequences we have assigned to Leptolyngbyoideae could come from *Planktolyngbya* or even *Pseudanabaena*. Two sister clades, *Planktothrix rubescens* and *P. agardhii*, had relatively high statistical support and we were able to assign three sequences to *P. agardhii* and two to *P. rubescens*. Interestingly, *P. agardhii* was identified in microscopic analyses, while *P. rubescens* was not.

**Figure 3:**
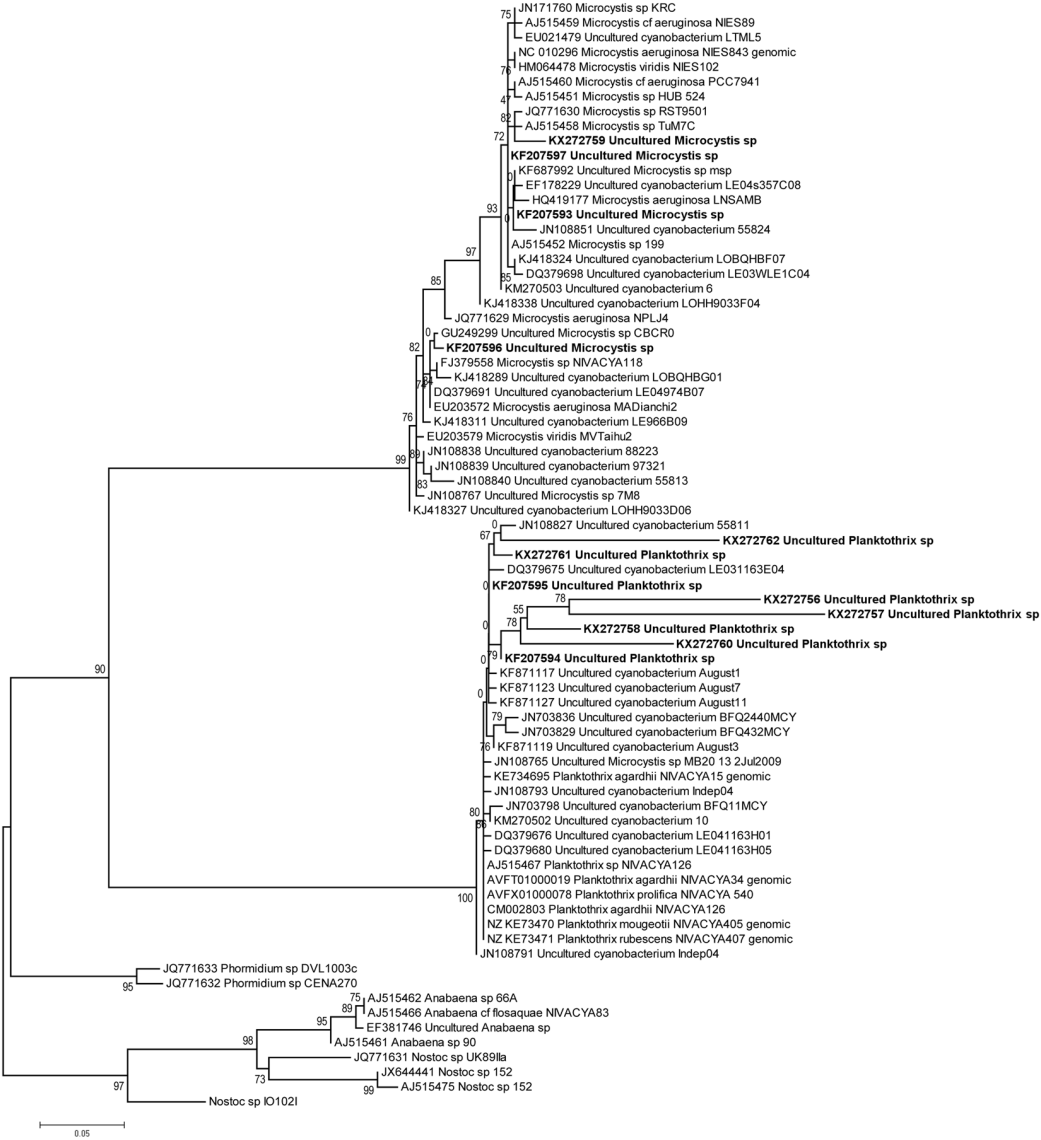
Phylogenetic tree obtained with *mcy*A gene fragments corresponding to the sequences obtained from DGGE bands. Numbers at nodes represent branch frequencies. Evolutionary trees, involving 75 sequences, were estimated by the PhyML program^49^, using the Maximum Likelihood method based on the General Time Reversible model with a discrete Gamma distribution model for evolutionary rate differences among sites (4 categories (+G, Gamma shape parameter = 1.602)). Branch frequencies were calculated by aLRT test, according to Anisimova and Gascuel^50^. The tree with the highest log likelihood (−2148.459) is drawn to scale, with branch lengths measured in the number of substitutions per site. The rate variation model allowed for some sites to be evolutionarily invariable; however, no such sites were revealed (+I, Proportion of invariant sites = 0.000).

Strikingly, the phylogenetic analysis of DGGE-derived *mcy*A sequences revealed much less variation. Only two genera: *Planktothrix* and *Microcystis* (Fig. 3) were identified. Altogether, eight different *Planktothrix* and four *Microcystis* sequences were identified. However, we were not able to assign them to a particular species of *Planktothrix* or *Microcystis* based on phylogenetic analysis of *mcy*A gene fragments.

## MC toxins by ELISA, HPLC and LC-MS

### ELISA: MC concentrations in water, and total MC concentrations

Low concentrations of microcystins were detected in the studied lakes by ELISA during 2012 and 2013, despite the presence of all the three *mcy* genes in all of the lakes (Tables S2 and S4). The concentrations of MCs in 2012 varied from 0.1 µg L^−1^, which was at the detection level for the method, to 0.3 µg L^−1^. In 2013, the concentrations of microcystins in filtered water varied from 0.2 to 0.6 µg L^−1^. Analysis of microcystins in unfiltered water, performed after the sonication of cells, showed that total concentrations of MCs were from 2 to 20 times higher than those dissolved in water. Using ELISA, we did not detect even trace concentrations of microcystins in Lake Mamry, despite the presence of *mcy*-genes in most of the samples in this lake (except in May 2011). In September 2013, we did not detect microcystins dissolved in the water, only MCs present in the cells, with the exception of Lake Mikołajskie, in which the concentration of MCs in the water was 0.1 µg L^−1^ and the total concentration reached 2.1 µg L^−1^. These were also the highest concentrations measured by ELISA during the entire study (Table S4).

### HPLC and LC-MS/MS

HPLC-DAD analysis of cyanobacterial material collected in 2012 and 2013 from the Great Masurian Lakes did not reveal the presence of cyanotoxins. Positive results were obtained when samples were analyzed using the LC-MS/MS method characterized by higher sensitivity. Altogether, seven MC variants were identified, with [Asp^3^]MC-RR being the most frequent (Table S5). In 2012, MCs were detected in five of the eight lakes. In Mikołajskie Lake, [Asp^3^]MC-RR was found in samples collected from May to August 2012. This demethylated microcystin was also present in samples from Lake Kisajno (in May and August), Lake Bełdany (in May and August) and Lake Śniardwy (in May).

The LC-MS/MS analyses of cyanobacterial samples collected in 2013 showed the presence of MCs in eight out of nine studied water bodies. No toxin was detected in Lake Mamry. In the eight lakes, the toxins were found almost in all samples, with the exception of samples collected in May from Lakes Tałtowisko and Tałty. Also, in that year, the [Asp^3^]MC-RR variant was the most common and was identified in all lakes for which positive results were obtained (Table S5).

### Results of qPCR in environmental samples – the proportion of toxicity genes: *mcy*B vs. PC-IGS for *Microcystis*, and *mcy*A vs. 16S rRNA genes for *Planktothrix*

**Figure 4:**
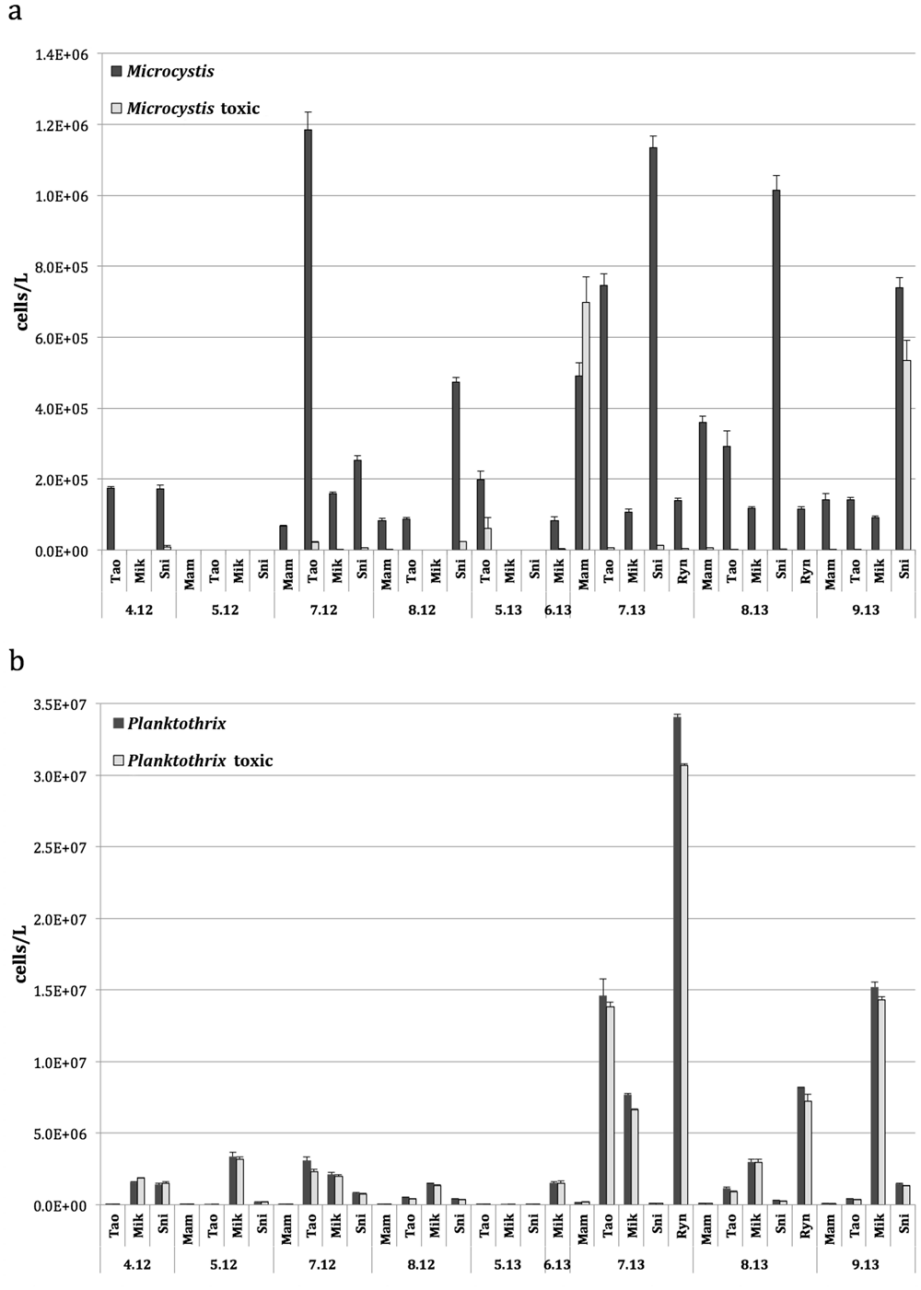
The total number of potentially toxic taxa – *Microcystis* (**a**) and *Planktothrix* (**b**), and the number of their toxic genotypes in the studied Great Masurian Lakes in 2012 and 2013, based on real-time PCR analyses. Mam – Lake Mamry, Tao – Lake Tałtowisko, Mik – Lake Mikołajskie, Ryn – Lake Ryńskie, Sni – Lake Śniardwy. Numbers denote month and year of the sampling.

The qPCR analysis with hydrolysis probes showed that in the community of cyanobacteria, the number of *Planktothrix* cells calculated on the basis of a fragment of the 16S rRNA gene varied between 3.8 × 10^3^ and 3.4 × 10^7^ cells L^−1^ (Fig. 4a). In 2012, the highest numbers of cells were noted in May in Lake Mikołajskie, and in July in Lake Tałtowisko. In 2013, the highest abundance of *Planktothrix* cells was noted in July in Lake Ryńskie, and very high in July and September, in Lakes Tałtowisko and Mikołajskie, respectively. The mean cell number of *Planktothrix* was 3.1 × 10^6^ cells L^−1^. The analysis of the *mcy*A gene showed similar values and the number of *mcy*A-possessing cells varied between 3.1 × 10^3^ and 3.1 × 10^7^ cells L^−1^, with a mean of 2.9 × 10^6^ cells L^−1^. The highest numbers of cells with *mcy*A genes were detected in the same lakes and on the same dates as in the case of *Planktothrix* cells based on the 16S rRNA gene. Although the maximal numbers of *Planktothrix* cells and *Planktothrix* cells with toxicity genes were higher in 2013 than in 2012 the mean values did not differ statistically. In the case of *Microcystis*, the total cell number varied between 0.0, when the *Microcystis* cells with PC-IGS genes were not detected by qPCR analysis, and 1.2 × 10^6^ L^−1^, when the mean cell number during the study period was 2.6 × 10^5^ L^−1^, which is more than 10 times less than in the case of *Planktothrix*. The number of *mcy*B gene copies varied from 0.0 up to 7.0 × 10^5^ L^−1^, but occasionally *mcy*B was not detected when PC-genes of *Microcystis* were found. Similar to *Planktothrix*, neither the total number of cells nor the number of cells with toxicity genes differed significantly during these two study years. The highest total number of *Microcystis* cells was noted in Lake Tałtowisko in July 2012, with very high numbers being found in Lake Śniardwy in July and August 2012. Unlike *Planktothrix*, the highest number of *Microcystis* cells with *mcy*B genes was not detected with the maximum total of *Microcystis* cells, but it was detected in Lake Mamry in July 2012 (Fig. 4b).

Detailed analysis of each sample showed that the share of cells bearing *mcy*A-genes in the community of *Planktothrix* varied between 75 and 100%, with a mean value of 91% ± 8.5 (Fig. S4). Also, the mean percentage of cells with *mcy*A genes calculated for each of the study years was similar: 90 and 91% respectively. In each of the four lakes analyzed by qPCR (Mamry, Tałtowisko, Mikołajskie and Śniardwy), the percentage reached 100% at least once in each sampling season. The highest share was noted in all four lakes in the spring, in April or May, and the mean share in the spring for all the studied lakes was 97%. The lowest percentage was noted in Lake Tałtowisko in July 2012, and in Lake Mamry in August and September 2013. The mean shares for summer and autumn were 88 and 86%, respectively.

The share of toxic genotypes in the case of *Microcystis* varied widely, between 0 and 100% (Fig. S4), but on average it was only 9% ± 24, with a mean share of 2% in 2012, and 13% in 2013. We did not find any seasonal patterns in the share of *Microcystis* cells with *mcy*B genes in the total *Microcystis* cell number. The share varied irregularly in each lake, reaching seasonal maxima in May, July or September.

We also compared the number of *Planktothrix* and *Microcystis* cells bearing *mcy*-genes with counts of all cyanobacterial cells obtained in microscopic analyses. This share was very low in both years; in 2012, the summarized share of ‘toxic’ *Planktothrix* and *Microcystis* in the total number of cyanobacteria varied between 0.01 and 0.80%, and in 2013 between 0.02 and 5.89%. The mean values were 0.18 and 1.42%, respectively.

### Relationships between environmental parameters and the occurrence of *mcy*-genes and MCs

**Figure 5:**
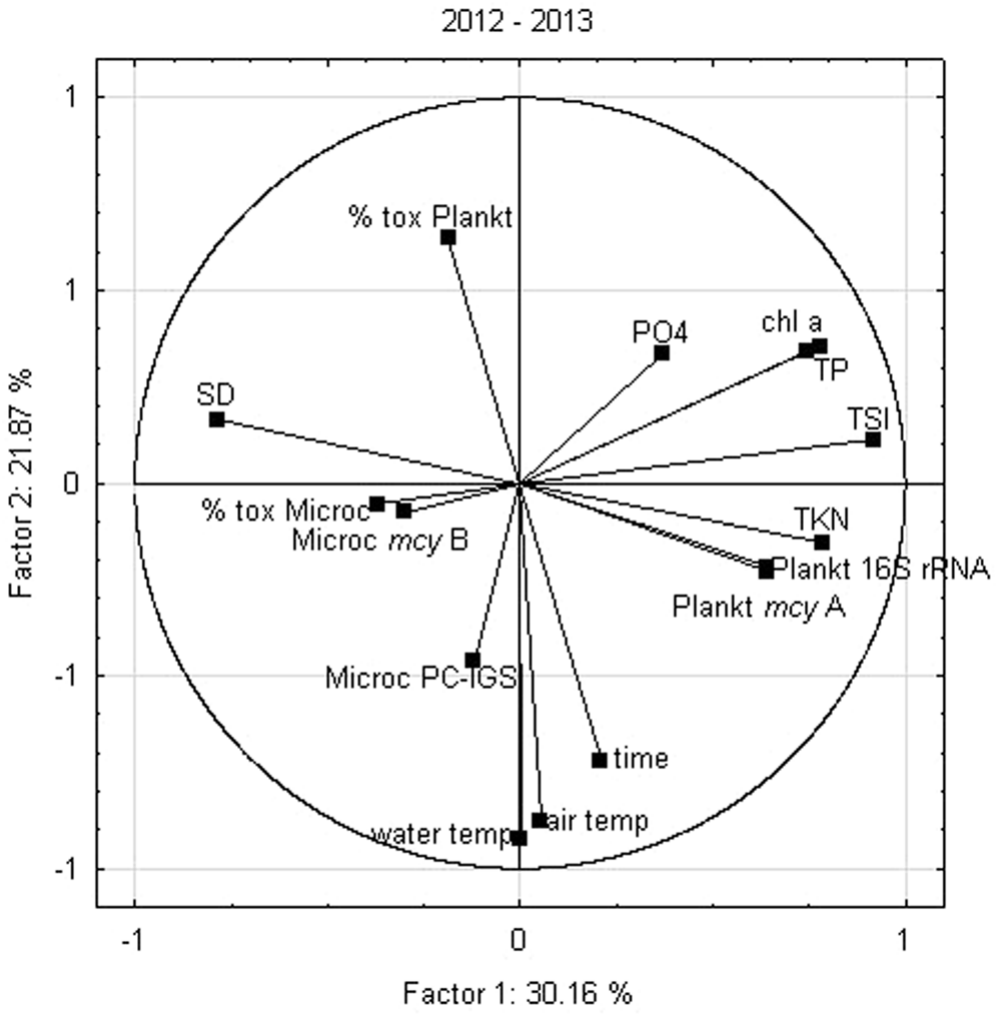
Principal component analysis (PCA) (Factors I and II) made on the loadings of environment variables and cyanobacterial parameters. SD – Secchi depth, time –month of sampling, air temp – air temperature, water temp – water temperature, chl *a* – chlorophyll *a*, TSI – Trophic State Index, TP – total phosphorus, PO_4_ – orthophosphates, Microc PG-IGS – total *Microcystis* (PC-IGS), Microc *mcy*B – potentially toxic *Microcystis* (*mcy*B), % tox Microc – share of potentially toxic *Microcystis* in total number of *Microcystis*, Plankt 16S rRNA – total *Planktothrix* (16S rRNA gene), Plankt *mcy*A – potentially toxic *Planktothrix* (*mcy*A), % tox Plankt – share of potentially toxic *Planktothrix* in total number of *Planktothrix*.

The PCA showed that Factor 1 was responsible for 30.2% and Factor 2 for 21.9% of the variability (Fig. 5). Total cell number of *Planktothrix* (16S rRNA gene) and the number of *Planktothrix* cells with *mcy*A correlated positively with each other and with TSI and total Kjeldahl nitrogen (TKN), as well as negatively with Secchi depth (SD). The percentage of *Planktothrix* cells with *mcy*A correlated negatively with month of sampling, and air and water temperatures, while the total numbers of *Planktothrix* cells and *Planktothrix* cells with *mcy*A-genes were independent of temperature and time. Total *Microcystis* cell numbers (based on PC-IGS) correlated slightly with water temperature, while *Microcystis* cell numbers bearing toxicity genes (*mcy*B), and the percentage of these cells in total *Microcystis* (PC-IGS) did not show a correlation with any of the factors or environmental variables.

Spearman’s rank order correlation analysis revealed several significant (*P* < 0.05) relationships between the occurrences and share of potentially toxic taxa with microcystins (not analyzed in PCA), and confirmed relationships with environmental data (Table S9). The concentrations of MCs in the water, and total concentrations of MCs (in water and intracellular) measured by ELISA correlated positively with the total population of the *Planktothrix* community, based on qPCR, and with the number of *mcy*A-carrying cells. Interestingly, the MC concentrations (in the water and in total) did not correlate with the percentage of cells with *mcy*A in the total number of *Planktothrix* cells, but they were positively correlated with the share of the total number of *Planktothrix* cells (based on 16S) and of *Planktothrix* with the *mcy*A gene in the total number of cyanobacteria. Additionally, we found that total MCs correlated positively with TP (total phosphorous) and TKN, while total MCs correlated negatively with SD (Table S9). Air temperature significantly positively correlated with the numbers of *Planktothrix* from microscopic counts, while SD correlated negatively. The total *Planktothrix* cell number, based on qPCR and number of cells with *mcy* genes (qPCR-*mcy*A), correlated positively with the lakes’ TSI and chlorophyll *a*, and negatively with SD. The percentage of cells with *mcy*A in the total *Planktothrix* cell count was negatively correlated with month of sampling, the temperature of the air and water, and with NH_4_^+^. *Microcystis* cell numbers obtained from qPCR analysis correlated positively with the temperature of the water.

## Discussion

The presented results show that all studied Masurian lakes host potentially toxic cyanobacteria. Microscopic analysis revealed that 22, out of 44 identified cyanobacterial taxa, belonged to potentially toxic taxa, including 16 potentially microcystin-producing. Two of them – *P. agardhii and M. aeruginosa* – are the most common and potent microcystin producers among freshwater phytoplankton^16^.

Contrary to the high diversity of cyanobacteria occurring in these lakes (identified microscopically and by ITS-DGGE), the DGGE analysis of *mcy* genes revealed that only *Planktothrix* and *Microcystis* carried the *mcy*A genes. Altogether, we identified 13 different *mcy*A DGGE sequences, of which we assigned eight to *Planktothrix* and five to *Microcystis*, but the phylogenetic analysis did not allow us to identify the species of these potential MC producers.

The possible reason for not detecting *mcy*A genes in other species is that the primers we used turned out not to be universal. They were designed for main microcystin producers, *Microcystis*, *Planktothrix* and *Dolichospermum* ^17^ and were considered universal^18^. Some authors using these primers for environmental samples were able to amplify the *mcy*A gene fragment only from *Microcystis* and *Planktothrix* ^13, 15^. From other cyanobacterial taxa such as *Dolichospermum* (*Anabaena*)^17, 19–21^, *Nostoc* ^19^, *Leptolyngbya* ^22^ and *Cylindrospermopsis raciborskii* ^23^, *mc*yA sequences were obtained only from cultivated strains, except for one case of *Nostoc* isolated from a lichen^24^. The other possible reason we were not able to recognize the diversity of *mcy*-bearing cyanobacteria is that the *mcy*A sequences are not known for all taxa. The GenBank database includes *mcy*A sequences belonging to only ten genera: *Dolichospermum* (*Anabaena*), *Cylindrospermopsis*, *Geitlerinema*, *Leptolyngbya*, *Mastigocladus*, *Microcystis*, *Nostoc*, *Phormidium*, *Planktothrix*, *Radiocystis*, of which only three genera (*Dolichospermum* (*Anabaena*)*, Microcystis* and *Planktothrix*) were found in the microscopic analyses of samples from GML. Thus, the low diversity of *mcy*A genes in GML is likely to be an underestimation, and we cannot rule out that, apart from *Planktothrix* and *Microcystis*, other potential microcystin producers were present there. However, in order to prove this, new primers, which would amplify the *mcy*A-gene fragments also of other taxa from environmental samples, should be designed.

During three years, the studied lakes were characterized by considerably high TSI (between 47 and 60) and chlorophyll *a* concentrations often exceeding or close to 20 µg L^−1^, which is considered the threshold level needed to classify the amount of phytoplankton as a bloom^25^. The mean share of cyanobacteria in the total phytoplankton biomass varied largely in the studied lakes, from 3 ± 1% to 70 ± 22% of the total phytoplankton biomass (Table S1). In our study, the *mcy* genes were accompanied by very low concentrations of microcystins (Table S4). Previous studies conducted in the GML system pointed to the presence of considerably high concentrations of microcystins (up to 12 µg L^−1^) in four lakes of the system^14^. [Asp^3^, dhb^7^]MC-RR was the most frequent microcystin variant in studied lakes, with [Asp^3^, Mdha^7^]MC-RR and MC-RR occurring more often than the other four variants found. [Asp^3^]MC-RR is known to be widely distributed in European bodies of water, including many Polish lakes^12, 26–28^ and frequently associated with *P. agardhii* populations^16, 29^. This variant was also detected by Mankiewicz *et al*.^14^ in four lakes within the GML system. Recently, [Asp^3^]MC-RR was detected in 11 out of 21 studied water bodies in Poland, and all 11 lakes were dominated by *P. agardhii*. *P. rubescens*, on the other hand, was found in only 3 of 238 studied lakes in Poland^27^. Populations of *P. rubescens* are also mostly characterized by production of [Asp^3^]MC-RR^16^.

According to Kosol *et al*.^30^ MC-containing strains of *P. agardhii* and *P. rubescens* usually produced 1–2 microcystins, including one of the demethylated MC-RR variants that were found also in our samples. Although the two species of *Planktothrix* analyzed by these authors differed with respect to pigmentation, the production of chlorophyll *a* and anabaenopeptins’ per biomass, no significant differences in the MC profile or content of the toxins were observed, which would allow for differentiation between the species. Suda *et al*.^31^ reported that the similarity of 16 S rRNA gene sequence and fatty acid composition in *P*. *agardhii* and *P. rubescens* were also high. Analysis of *Planktothrix* strains from Lake Steinsfjorden in Norway revealed that representatives of both species could be classified as the same ecotypes and chemotypes, suggesting significant overlapping of the two species^32^. Some strains from the two species can also belong to the same *mcy*B genotypes, although genotypes consisting exclusively of one species were also recorded. These controversies regarding classification were attributed to frequent recombination events^33^.

In our case, *Planktothrix*, identified microscopically as *P. agardhii*, occurred in all studied lakes, although the proportion of this cyanobacterium in the total number of cyanobacterial cells was very low, and the number of *Microcystis* was even lower. The very low biomass of identified potentially toxic taxa in the overall cyanobacterial population, and the even lower percentage in the total phytoplankton biomass, may explain the very low concentrations of MCs in the studied lakes.

*Planktothrix agardhii* is known as a potent toxin producing cyanobacterium that dominates in many eutrophicated shallow lakes^12, 26, 34^, but can also occur in higher numbers in deep meso-eutrophic and deep eutrophic lakes^7, 35^. While studying phytoplankton in the GML system in the 70 s, 80 s and 90 s, Spodniewska^6, 7, 36, 37^ noted very high numbers and biomass of *P. agardhii* in the studied lakes. Massive blooms of *P. agardhii* were reported in 1991–1993 in Lake Niegocin, the lake included in the study^8^. At that time, Lake Niegocin was highly eutrophicated, but with the modernization of the water treatment plant in Giżycko in 1995, the trophic status of the lake gradually decreased^38^, as did the phytoplankton biomass and share of cyanobacteria in it^8, 35^. However, recent results suggest that the process of oligotrophication has slowed down, if not stopped, especially in the central and southern part of the GML system^10, 38^. In this respect, we cannot rule out a repeated increase of *Planktothrix* biomass in the coming years.

The qPCR analysis revealed that the population of *Planktothrix* present in the four lakes studied by this method was almost entirely toxic, and the number of all *Planktothrix* cells and toxic cells correlated with concentrations of microcystins (Table S9).

The high share of toxic genotypes in *Planktothrix* populations in GML is an interesting phenomenon, because studies elsewhere in Europe demonstrate that the proportion of toxic genotypes in *P. agardhii* is usually strikingly lower^39^. Such a high portion of toxic genotypes is characteristic in turn of the phycoerythrin-rich *P. rubescens*. Kurmayer *et al*.^39^ reported a very high proportion of toxicity genes in populations of *P. rubescens*, which dominated in deep and mesotrophic lakes. Analyzing PC-IGS and microcystin-encoding genes, these authors divided *Planktothrix* into two lineages: one containing green-pigmented strains, which lacked toxicity genes, and a second containing red-pigmented and green-pigmented strains. Within the second lineage, the green strains were characterized by a low percentage of genotypes with *mcy*-genes, while the red strains were composed almost solely of genotypes bearing *mcy*-genes^39^. Further study carried out in lakes, varying in depth, trophic status, and the presence of green (PC-rich) and red (PE-rich) *Planktothrix*, confirmed this division and the differences between these two lineages of *Planktothrix* ^12, 33^. The authors reported that the percentage of toxicity genes in populations of green strains occurring in shallow lakes varied between 7 and 40%, while for red strains occurring in deep, stratified, mesotrophic lakes, it was between 75 and 100%. The same study showed that the green strains occurring in deep, stratified lakes were also characterized by much lower proportions of *mcy* genes than red strains from deep lakes, and this share did not differ significantly from that noted for green strains in shallow lakes^12^. The presence of these two main lineages of *Planktothrix*, and a third lineage, consisting of green-pigmented strains from tropical regions, was recently confirmed in 138 strains and it was shown that MC and other peptide production depends on *Planktothrix* phylogeny and ecophysiological adaptations^40^.

The results of our study are relevant because the *Planktothrix* occurring in our lakes, which are stratified and eutrophic, contained almost 100% of toxic genotypes, which makes the population more similar to the red *P. rubescens*, thriving in deep, mesotrophic lakes, than to other green *Planktothrix*, characteristic of eutrophic, often shallow lakes. The phylogenetic affiliation of two DGGE-ITS sequences to *P. rubescens* agrees with the high share of toxicity genes. There is, however, an inconsistency with the pigmentation of this *Planktothrix* closely related to *P. rubescens*. In our study, we have not analyzed the phycocyanin and phycoerythrin operons. In microscopic analyses, this *Planktothrix* appeared green, and isolation of filamentous cyanobacteria did not produced red *Planktothrix* strains. Typical *P. rubescens* has also not been detected in earlier studies^6–8, 36, 37^. Therefore, our results indicate the presence of a strain of *Planktothrix* in GML, which, based on ITS analysis, is closely related to *P. rubescens* and is characterized by a very high share of toxicity genes, typical for *P. rubescens*. However, like *P. agardhii*, it is green and thrives in the epilimnion of these eutrophic lakes.

The hypothesis of Tooming-Klunderud *et al*.^41^ may shed some light on the subject. They analyzed the whole genomes of eight *Planktothrix* strains (four red and four green) isolated from two lakes in Norway. They found that strains of the same chemotype, producing the same oligopeptides, were closely related regardless of color. Furthermore, the authors suggested that the ancestral *Planktothrix* was green and obtained the whole PE gene cluster by horizontal gene transfer, similarly to *Synechococcus* ^42^. Based on phylogenetic analyses of the 16S rRNA gene and the PC-IGS operon, Jasser *et al*.^43^ hypothesized that strains of *Synechococcus* from one of the clades (clade M) present in GML were originally green. They proposed that subsequently, during the oligotrophication of these glacial lakes, the strains could have acquired red pigmentation from closely related, ubiquitous, PE-rich strains from another clade (clade B), which allowed them to adapt better to the changing environment. These two hypotheses could explain the nature of the green color in the *Planktothrix* occurring in GML closely related to *P. rubescens*.

Another phenomenon is the decrease in the proportion of genotypes containing *mcy*A genes in the summer. The share of toxic genotypes in *P*. *rubescens* is stable during the vegetation season and is close to 100%^12, 44^. This stability makes it possible to predict whether the cyanobacterial blooms, if they occur, will be toxic. We noted herein a slight, though statistically significant, decrease in the share of toxic genotypes in the summer, when the air and water temperature was higher. Unfortunately, we could not differentiate between *P. agardhii* and *P. rubescens* by qPCR. We can, however, hypothesize that the decrease of the percentage of cells with toxicity genes was the consequence of a growing second population of *Planktothrix*, a typical, green *P. agardhii*, characterized by no, or a low percentage of toxicity genes. *P. agardhii* was shown to take advantage of the increased temperature, in which it may even outcompete *P. rubescens* ^45^. In line with this, the PCA analysis in our study showed a negative correlation between the proportion of cells with toxicity genes, and the air and water temperature. This should be, however, further investigated, e.g. by using primers that differentiate between these two species or by metagenomic analysis of 16S rDNA and specific microcystin-encoding gene amplicons.

The statistical analyses (Spearman’s rank-order correlation) revealed that the abundance of *Planktothrix* and toxic *Planktothrix* genotypes correlated with concentrations of MCs in water, and total concentrations of MC (intracellular and in water). This finding suggests that *Planktothrix* was the main toxin producer in GML. The correlations of the total number of *Planktothrix* cells, as well as *Planktothrix* cells with toxicity genes to TKN and TSI, suggest that potential MC toxicity in the studied lakes may increase along with their further eutrophication. Considering the massive blooms of *Planktothrix* recorded in the 1980s and 90s, when the lakes were more eutrophicated, such blooms are possible in the future if the trophic status of the studied lakes increases again^38^. The Spearman’s rank-order correlation analysis demonstrated that MC concentrations correlated with the total number of *Planktothrix* cells and the number of cells with *mcy*A genes, but not with the proportion of ‘toxic’ cells. The latter result is rather puzzling, though it could be explained by the fact that the concentrations of MCs in the studied lakes were low. Thus, it is plausible that the percentage of cells with toxicity genes was above a threshold level when the concentrations of toxins became measurable, but the concentrations were too low to trace the relationships with the percentage of toxic genotypes. However, the results suggest that the very high proportion of genotypes containing *mcy*A genes in the *Planktothrix* population in GML during most of the season makes it possible to predict whether the bloom, if formed by *Planktothrix*, will be toxic. It also seems that the slight decrease in the share of ‘toxic’ genotypes noted in the summer does not exclude the possibility of forecasting it.

Based on present results, we cannot predict whether the bloom of *Microcystis*, if it occurs, will be toxic. We did not find any trends in the proportion of toxic genotypes within the total *Microcystis* cell number over time, or any significant relationships between *Microcystis* cells with and without *mcy*B genes, and environmental variables. However, as the numbers of *Microcystis* detected in this study by microscopic and qPCR analyses were low, it is possible that they were below the detection level for both methods. Also other studies suggest that changes in the share of toxic *Microcystis* genotypes are too irregular^46, 47^, and therefore, reliable prediction of toxic *Microcystis* blooms is difficult. On the other hand Kurmayer and Kutzenberger^48^ found the proportion of toxic genotypes in *Microcystis* to be very stable during the entire season, which could make such forecasting possible. However, Bevendorf et *al*.^13^ concluded that the microcystin-encoding genes are in general not good indicators of toxin occurrence in the environment, and that new studies should concentrate on measuring toxin gene expression.

## Conclusions

Our study suggests that *Planktothrix* is the main genus responsible for microcystins occurring in the Great Masurian Lake system. Based on the results of qPCR analysis, we can predict that, if there were to be a *Planktothrix* bloom in GML, it would be toxic. We propose that this analysis is a good, and considerably easy, tool to predict the probability of the bloom being toxic not only in GML, but also in other waters in which *Planktothrix* occurs. The study also revealed that to detect potentially microcystin-producing species – other than *Planktothrix* and *Microcystis* – new primers for *mcy*A-gene should be designed for the environmental studies, as the ones, which were used routinely up till now are not universal.

As concerns the Great Masurian Lake, we expect that further eutrophication of the system may lead to an increase in *Planktothrix* biomass, as was already noted in the 1970s, 80s and 90s, and therefore the threat of a rise of toxic *Planktothrix* blooms in the GML system cannot be ruled out. However, because it is not clear which genotypes would increase along with the eutrophication of these lakes, it is important to regularly monitor the lakes with respect to the presence of potentially toxic genotypes that could be classified as *P. rubescens* or *P. agardhii*.

## Materials and Methods

### The study area and sampling

The study was conducted in The Great Masurian Lake system (GML), located in the North-Eastern part of Poland (54° N and 22° E). The lakes are connected with natural and man-made canals, and are spread North-South for over 100 km (Fig. 1). They are of glacial origin; three of them are moraine-dammed lakes, including the two largest lakes in Poland: Śniardwy and Mamry. The other five sampled lakes represent ribbon-type lakes. All the studied lakes are considerably deep, with summertime thermal stratification, which lasts from June to October. The lakes vary in trophic status from mesotrophic to highly eutrophic (Table S1), and are characterized by rich and mixed cyanobacterial communities^9^. In 2011, 2012 and 2013, eight lakes were sampled four times during the vegetation season: in the spring (April and/or May) before stratification, during the summer (July and August), and in the early autumn (September). Integrated samples were collected with a 2-L Limnos sampler at 0.5 m depth intervals, from the euphotic zone during the mixing period, and from the epilimnion during the summer stratification period.

Abiotic parameters characterizing the ecosystems were determined during each sampling in all the lakes, and the chlorophyll *a* concentration was measured. The entire phytoplankton community was analyzed in 2011 and 2012. In 2013, cyanobacterial communities were analyzed microscopically in the four lakes, which were studied by qPCR, in order to assess the share of cyanobacterial strains bearing *mcy* genes in the total number of cyanobacterial cells. The diversity of cyanobacteria was studied using DGGE analysis of the 16S-ITS gene fragment and the diversity of potentially toxic taxa based on a highly conserved domain in the *mcy*A gene. The sequences obtained from DGGE bands were used to build phylogenetic trees with 16S-ITS and *mcy*A gene reference sequences.

Four lakes (Mamry, Tałtowisko, Mikołajskie and Śniardwy) were sampled four times in 2012 and in 2013, in order to assess the percentage of toxicity genes (*mcy*A and *mcy*B) in the given taxon and in the overall communities of cyanobacteria. Lake Ryńskie, a highly eutrophic lake, was additionally sampled in July and August 2013 for qPCR analyses, and Lake Mikołajskie in June 2013. All methods are described in detail in the Supplementary Information.

## Electronic supplementary material

Supplementary Information
