# Supplementary material for: Predicting blooms of toxic cyanobacteria in eutrophic lakes with diverse cyanobacterial communities
Source: Sci Rep. 2017 Aug 21;7:8342. doi: 10.1038/s41598-017-08701-8 (PMC5566422; doi:10.1038/s41598-017-08701-8)
Supplement: Supplementary file 1 — Supplementary Information [file 41598_2017_8701_MOESM1_ESM.doc]

**Predicting blooms of toxic cyanobacteria in eutrophic lakes with diverse cyanobacterial communities**

Bukowska Aleksandra, Kaliński Tomasz, Koper Michał, Kostrzewska-Szlakowska Iwona, Kwiatowski Jan, Mazur-Marzec Hanna & Jasser Iwona

**Supplementary information**

**Methods**

**Basic physical, chemical and biological analyses.** The temperature profile, oxygen concentration, conductivity, turbidity, chlorophyll *a* fluorescence, epilimnion thickness and thermocline depth was determined using a multiparameter probe – YSI 6000 (YSI Inc., Yellow Springs, USA). Additionally, the Secchi depth (SD) was measured. The concentrations of basic nutrients: such as PO_4_-P – orthophosphates, NH_4_^+^ – ammonium, NO_3_^-^ – nitrate, TKN – total Kjeldahl nitrogen (NH_4_^+^-N org), as well as TP (total phosphorus) and TN (TKN+N-NO_3_) were analyzed according to standard methods^1−4^. Chlorophyll *a* (Chl *a*), corrected for pheopigments, was analyzed fluorometrically (TD-700, Turner Biosystems USA) after extraction with 98% acetone from phytoplankton cells, from 10-20 mL of lake water, collected on GF/F Whatman filters (25 mm filter diameter), following the procedure developed by Arar and Collins^5^. The mean trophic state index (TSI) for every studied lake at each sampling time was calculated on the basis of chlorophyll *a*, total phosphorus concentrations (TP), and Secchi depth (SD) water transparency, according to Carlson^6^. The TSI from each sampling time and place was used in the statistical analyses. In Table S1, we present the mean spring and mean summer values of the given parameters.

Water samples (500 mL) were fixed with Lugol solution and additionally with 37% buffered formaldehyde, to a final concentration of 1.5%. The samples were stored in the dark at 4 °C and, within a few months, were analyzed using the Utermöhl method^7^. Samples of 5 to 50 mL were analyzed in triplicate (unless the results from two analyses were very similar) with an inverted microscope (NIKON Eclipse TS100) under 100, 200 and 400 × magnification. At least 400 cells or 20 fields were counted. Phytoplankton biomass was calculated on the basis of the biovolume of cells, assuming that 10^6^ µm^3^ equals 1 µg of fresh biomass^8^. The biovolume of cells was estimated according to equations provided by Hutorowicz^9^ and Wetzel and Likens^10^.

**Counting cells from culture strains.** qPCR standard curves were determined using culture strains of *Microcystis aeruginosa* (CCNP 1103, University of Gdansk Culture Collection), and *Planktothrix agardhii* (6.89, SAG The Culture Collection of Algae at the University of Göttingen, Germany), which have the *mcy*-gene cluster in their genomes. Strains were grown nonaxenically, in sterile conditions in BG11 medium^11^. Cells were harvested during the stationary phase and their densities were determined immediately before DNA isolation, using the epifluorescent microscope Nikon Eclipse with a narrow banded CY3 filter in a green light at 1000 × magnification. In the case of *Microcystis aeruginosa*, individual cells were counted, while in the case of *Planktothrix agardhii,* the total length of filaments was measured and then divided by the average length of a *Planktothrix* cell, which in this study was 4 µm.

**DNA extraction from cultures and environmental samples.** A total of 250 mL of lake water was filtered onto 0.2 µm Nuclepore polycarbonate filters (47 mm in diameter, Whatman Schleicher & Schuell) and stored at -80 °C for later DNA extraction. DNA from frozen filters was purified using the GeneMATRIX Soil DNA Purification Kit, EURx, according to the manufacturer’s instructions. DNA from 1 mL culture strains was isolated using the GeneMATRIX Bacterial & Yeast Genomic DNA Purification Kit, according to the manufacturer’s instructions. In both protocols, in the last step, DNA was suspended in 50 µL of elution buffer. The quantity and quality of isolated DNA was assessed by agarose electrophoresis, as well as by fluorometric methods, using Quant-iT dsDNA Assay Kit, Broad Range (Q33130, Life Technologies), and by microplate reader Synergy H1 (BioTek).

**Amplification of *mcy*A, *mcy*D and *mcy*E genes from environmental samples.** Preliminary monitoring of the presence of toxic genotypes in environmental samples, conducted in 2011 and 2012 (Table S2), was performed by amplifying three fragments of the *mcy*-gene cluster, using the following primer pairs: for *mcy*A, mcyA-Cd1F (5’-AAAATTAAAAGCCGTATCAAA-3’) and mcyA-Cd1R (5’-AAAAGTGTTTTATTAGCGGCTCAT-3’)^12^; for *mcy*D, mcyDF (5’-GATCCGATTGAATTAGAAAG-3’) and mcyDR (5’-GTATTCCCCAAGATTGCC-3’)^13^; and for *mcy*E, mcyE-F2 (5’-GAAATTTGTGTAGAAGGTGC-3’) and mcyE-R4 (5’-AATTCTAAAGCCCAAAGACG-3’)^13^. Amplification was run in a Mastercycler epgradient S thermocycler (Eppendorf), using the Taq PCR Core Kit (Qiagen). The reaction mix (25 μL) contained approximately 20 ng of template DNA, 0.4 mM of each primer, 0.5 U Taq polymerase, 0.2 mM dNTPs, 4 mM MgCl2, 1× reaction buffer, 1× Q solution and deionized water. Cycling conditions were: for *mcy*A, preincubation at 94 °C for 5 min, 33 cycles of denaturation at 94 °C for 30 s, annealing at 56 °C for 30 s, elongation at 72 °C for 30 s; for *mcy*D and *mcy*E, 94 °C for 5 min, 30 cycles of 30 s at 94 °C, 30 s at 49.5 °C, 1 min at 72 °C.

**DGGE analysis.** In DGGE, we used two primer pairs: (GC)CSIF (5’-G(T/C)CACGCCCGAAGTC(G/A)TTAC-3’) and 373R (5’-CTAACCACCTGAGCTAAT-3’), targeting ITS fragment, for analyses of total cyanobacteria^14^; and (GC)mcyA-Cd1F (5’-AAAATTAAAAGCCGTATCAAA-3’) and mcyA-Cd1R (5’-AAAAGTGTTTTATTAGCGGCTCAT-3’), targeting a highly conserved domain in the *mcy*A gene, for analyses of potentially toxic cyanobacteria^12,15,16^.

A 40-nucleotide GC clamp (5’-CGCCCGCCGCGCCCCGCGCCCGGCCCGCCGCCCCCGCCCC-3’), which prevents the complete separation of two DNA strands in DGGE, was added to the 5′ ends of both forward primers. PCR reactions were performed in a Mastercycler epigradient S Thermocycler (Eppendorf, Germany) in a volume of 25 μL. The reaction mix contained approximately 20 ng DNA, 0.4 mM of each primer, Taq PCR Core Kit (Qiagen) – 0.5 U Taq polymerase, 0.2 mM dNTPs, 4 mM MgCl2, 1× reaction buffer, 1× Q solution and deionized water. Cycling conditions for ITS amplification were as follows: first 20 cycles – preincubation at 94 °C for 4 min; denaturation for 1 min at 94 °C; annealing for 1 min with an initial temperature of 62 °C, decreasing by 0.5 °C after every cycle to 52 °C (to reduce non-specific annealing of the primers), 1 min elongation at 72 °C; last 10 cycles – 94 °C for 30 s, 51 °C for 40 s, and 72 °C for 40 s; final step – 10 min at 72 °C. For *mcy*A amplification the conditions were as follows: pre-incubation at 94 °C for 4 min; first 20 cycles: 94 °C for 1 min, an initial annealing temperature of 59 °C decreasing by 0.6 °C in each cycle to 47 °C for 1 min; 72 °C for 1 min; last 10 cycles – 94 °C for 30 s, 47 °C for 40 s, 72 °C for 40 s; final step – 10 min at 72 °C^15^.

DGGE was performed in a BioRad ™DCode Universal Mutation Detection System on 1-mm-thick vertical gels, containing 7% (w/v) polyacrylamide (acrylamide/bisacrylamide, ratio 37.5:1.0), with the linear gradient of denaturants in the gel increasing from 20% to 60% (100% denaturant defined as 7 M urea and 40% (v/v) formamide). Electrophoresis was carried out in the TAE buffer at a constant temperature (60 °C), initially at 200 V for 5 min, followed by 16 h at 50 V. Gels were stained in a mixture of 14 mL 10,000×SYBR Green (Sigma-Aldrich, Cat No. S9430) and 200 mL deionized water for 15 min, viewed under UV light and photographed. Clearly visible bands were excised from the gel, placed in 40 μL of sterile deionized water and incubated at 4 °C for 24 h. The eluent was used as a template for reamplification with the same primer pair and run on another DGGE gel to confirm homogeneity. Then, the reamplified samples were Sanger-sequenced using the BigDye Terminator Cycle Sequencing Ready Reaction Kit (Applied Biosystems).

**Phylogenetic analysis.** Sequences obtained from DGGE bands were used as queries for Blast searches in GenBank (http://blast.ncbi.nlm.nih.gov/Blast.cgi). One hundred sequences with the lowest E-value were pooled for each sequence. All collected sequences were aligned using Muscle^17^, implemented in Mega 7^18^, and redundant sequences were removed from the alignment. *mcy*A DNA sequences were translated into protein sequences and aligned by Muscle. Obvious sequencing errors, resulting in frame shifting, were corrected. The Maximum Likelihood analysis was performed and GTR model parameters were estimated with the PHYML program^19^. Tree stability was estimated by the aLRT test^20^. All sequences used for phylogenetic analysis were deposited in the GenBank database. The accession numbers for ITS sequences are KF207558–KF207577, KF207579, KF207580, KF207582–KF207592, and for *mcy*A sequences, accession numbers are KF207593–KF207597, KX272756–KX272762.

**Microcystin analysis by ELISA in water and in cells of cyanobacteria.** Microcystin (MC) analysis was performed for filtered lake water samples, collected in 2012 and 2013 (Table S4), with the ELISA kit for microcystins: EnviroGard®Microcystins’7’ Plate Kit (New Castle, USA). The filtered samples were frozen and kept at a temperature of -25 °C. After thawing, the samples were analyzed following the protocol of the manufacturer. Additionally, in order to obtain total concentrations of MC, including in the cells of cyanobacteria, samples of unfiltered lake water from 2013 were frozen and sonicated with Ultrasonic Liquid Processing Vibra Cell VCX 1300, at 20 kHz frequency, pulse 30:60 s for 15 min after thawing. The samples were checked microscopically to ensure that the cyanobacterial cells were disrupted. The microcystins were then further analyzed, as in the case of microcystins dissolved in lake water.

**HPLC and LC-MS/MS.** Cyanobacterial material collected on Whatman GF/C glass microfiber filter discs was extracted with 90% methanol in water (1.5 mL). To disrupt the cells, a 10-min bath sonication (Sonorex, Bandeline, Berlin, Germany), followed by a 1-min probe sonication with an ultrasonic disrupter HD 2070 Sonopuls (Bandeline, Berlin, Germany), equipped with the MS 72 probe, was used. After centrifugation at 10,000 g for 15 min, the extracts were first analyzed with a HPLC equipped with a diode array detector (DAD) (Agilent 1200, Agilent Technologies, Waldboronn, Germany). The absorbance at 238 nm was monitored. Separation was performed on a Luna RP-18 (3.0 mm × 150 mm; 3 µm) column, kept at 30 °C. A gradient elution with a mixture of 5% acetonitrile in water (A) and 100% acetonitrile (B), both containing 0.05% trifluoroacetic acid, at a flow rate of 0.5 mL min^-1^, were used. In the case of microcystin standards (Alexis Biochemicals, Lausen, Switzerland), the detection limit was within the range of 0.1to 0.5 µg mL^-1^. The method was adapted and optimized in our laboratory and has been routinely used in several of our previously published works^21^.

In the next step, chemical analyses of microcystins in cyanobacterial samples were performed with the application of a mass spectrometer (QTRAP 5500, Applied Biosystems Sciex; Concorde, ON, Canada) coupled online with a liquid chromatography system (Agilent 1200, Agilent Technologies, Germany) as described in Šulčius *et al*.^21^. For toxin separation, a Zorbax Eclipse XDB-C18 column (4.6 × 150 mm; 5 µm) (Agilent Technologies, Santa Clara, California, USA) and a gradient elution with a mixture of 5% aqueous acetonitrile solution (A) and 100% acetonitrile (B), both containing 0.1% formic acid, were used. The profile of the produced microcystins was determined in information dependent acquisition mode (IDA), while for structure elucidation, enhanced ion product mode (EIP) was applied. Both types of experiments were conducted at positive ionization. Fragmentation spectra were collected with collision energy (CE) of 60 V with a collision energy spread (CES) of 20 V. Data acquisition and processing were accomplished using Analyst QS® 1.5.1 software. In the case of microcystin standards, the detection limit was within the range of 0.5 to 1.0 ng mL^-1^. The standards of the following microcystins were purchased from Enzo Life Sciences (Lausen, Switzerland) as Hepatotox SetTM1 (purity ≥ 95% HPLC): microcystin-LA, -LF, -LR, -LW, -LY, -RR, -YR. In addition, [d-Asp3]MC-RR, [d-Asp3]MC-LR and MC-WR were purchased from the same source. As was indicated in experimental part, the identification of the toxins was based on their product ion spectrum and MRM spectrum. In the case of toxins, whose standards were not available, the correlation between the concentration and peak area of microcystin with similar structure was taken into account in quantitative analysis. The results are presented in Table S5.

**qPCR protocols.** We followed the minimum information for publication of quantitative PCR experiments (MIQE) guidelines^22^. Four different Taq nuclease assays (TNAs) in two multiplex reactions were applied in these analyses. One qPCR assay quantified the total number of *Microcystis* by amplification of the PC operon fragment and the abundance of potentially toxic *Microcystis* by the *mcy*B gene fragment, using primers and probes designed by Kurmayer and Kutzenberger^23^ (which in this study was used in a multiplex reaction). In the second multiplex reaction, we used primers and probes designed by Ostermaier and Kurmayer^24^, and Briand *et al*.^25^, which quantify the total number of *Planktothrix* (16S rRNA gene fragment) and potentially toxic *Planktothrix* (*mcy*A gene fragment). Details of primers and probes used are in Table S6.

qPCR TNA reactions were carried out in the LightCycler 480 instrument with LightCycler 480 Software (Roche, Basel, Switzerland). All reactions were performed in triplicate, in white 384-well plates (FrameStar 4ti-0381, 4titude, Wotton, United Kingdom), in a volume of 10 µL. The multiplex reaction mix for *Microcystis* quantification contained: 5 μL Master Mix (LightCycler 480 Probes Master, Roche, Basel, Switzerland), 0.3 μM of primers and 0.1 µM of hydrolysis probe targeting the PC operon, 0.9 µM of primers and 0.25 µM of hydrolysis probe targeting the *mcy*B gene, and 2 µL of template DNA. The multiplex reaction mix for *Planktothrix* quantification contained: 5 µL Master Mix (LightCycler 480 Probes Master, product no. 04887301001, Roche, Basel, Switzerland), 0.3 µM of each primer, 0.2 µM of each hydrolysis probe and 2 µL of template DNA. The hydrolysis probes had a fluorescent reporter dye attached to the 5’ end, and a quencher attached to the 3’ end. We used 6-carboxyfluorescein (FAM), LightCycler Cyan 500 and LightCycler Red 610 dyes and black hole quencher 1 (BHQ-1), or black hole quencher 2 (BHQ-2) (Table S6). The qPCR program was adjusted to be the same for both multiplex reactions. This allowed us to perform all the TNA assays together on one plate. The qPCR reaction was initiated by a 10-min polymerase activation step, followed by 60 cycles of denaturation at 95 °C for 15 s, annealing and synthesis at 60 °C for 30 s, and 1 s fluorescence detection step at 72 °C.

**qPCR standard curves and detection limits.** For all four assays, standard curves were determined based on a microscopically predefined number of cells of *Microcystis* and *Planktothrix* strains. We used the approach described by Kurmayer and Kutzenbrger^23^, as well as Zhang *et al*.^26^. Standard curves were created from Cq values related to the number of cell equivalents in the qPCR reaction, rather than the number of gene copies, assuming that the gene copy number is constant in each cell.

All four five-fold dilution six-point standard curves were prepared from the same *Microcystis* and *Planktothrix* DNA mix, in which the concentration of DNA (in cell equivalents) was: 141,000 cells µL^-1^ for *Microcystis* and 112,000 cells µL^-1^ for *Planktothrix* respectively. Standard curves were calculated by performing linear regression analysis using LightCycler 480 Software (Roche, Basel, Switzerland), where the cells/reaction concentration values were on the logarithmic X-axis, and the Cq values were on the Y-axis (Fig. S1; Table S7). Quantification limits in the (MP) standard curves ranged from 282,000 to 90 cells/reaction for *Microcystis* and from 224,000 to 72 cells/reaction for *Planktothrix*. Obtained concentrations (cells/reaction) in the analyzed environmental samples were converted into the final cell density in 1 L of lake water.

**Specificity of the TNA and tests of inhibition.** The specificity of primers and probes used was confirmed in earlier publications^23,27^. Primers and probes used in *Microcystis* quantification assays were tested both *in silico* and experimentally in the presence of DNA of other cyanobacterial taxa (*Aphanizomenon*, *Limnothrix, Planktothrix agardhii*). The specificity of these TNA assays was confirmed only for *Microcystis*. The presence of DNA from other taxa had no effect on amplification efficiency^23^. The results obtained by Savichtcheva *et al*.^27^ showed that the primers and probes used in *Planktothrix* quantification assays were specific for this taxon, but not for other species of cyanobacteria (*Microcystis, Lyngbya, Synechococcus, Dolichospermum* (*Anabaena*)). The presence of DNA from these taxa had no significant effect on amplification efficiency.

In this study, we also performed several tests to confirm the specificity of the TNA assays in the presence of other cyanobacterial DNA and environmental backgrounds. We used a *Microcystis*-specific qPCR mix and, as a DNA template, various dilutions (36−112,000 cells µL^-1^) of DNA isolated from the *Planktothrix* strain. The same test was performed with a *Planktothrix-*specific qPCR mix and *Microcystis* DNA in various dilutions (45−141,000 cells µL^-1^) as a template. Specificity tests showed that the template DNA from the *Microcystis* strain does not give any qPCR results with the *Planktothrix* quantification mix, and also that the DNA isolated from the *Planktothrix* strain does not yield any results with the *Microcystis* primers and probes.

Furthermore, for each assay, two additional curves were established to test specificity and potential inhibition. The parameters of the main standard curves are in Table S7, and additional standard curves are in Table S8.

We checked whether mixing of *Microcystis* and *Planktothrix* DNA in the template used for standard curves affected quantification in the TNA assays. A series of five-fold dilutions of DNA were prepared at the same concentrations as in the MP standard curves, from the *Microcystis* and *Planktothrix* strains separately, and additional curves on the basis of these dilutions were created (labeled M and P standard curves).

We also verified whether the natural background in environmental samples (foreign DNA and possible inhibitory substances) affected the efficiency of the qPCR. For this purpose, other standard curves were determined (labeled MP+L), in which the template was the same DNA mix as in the MP main standard curves, but with the addition of environmental DNA at a concentration of about 5 ng µL^-1^ in the undiluted sample. Environmental DNA was isolated from lake Tałtowisko in September 2011. Microscopic analyses did not reveal the presence of *Microcystis* or *Planktothrix* in this sample, while other taxa of cyanobacteria such as *Snowella, Merismopedia, Chroococcus, Aphanocapsa, Aphanothece, Woronichinia, Dolichospermum, Pseudanabaena, Aphanizomenon, Cuspidothrix, Limnothrix* and *Planktolyngbya* were found. However, we cannot exclude the presence of trace amounts of *Microcystis* or *Planktothrix* in the sample. We also verified whether the results of quantification in the Masurian Lake samples differed depending on the variant of the standard curve used.

In all cases, the number of cells estimated using the MP standard curves correlated strongly (R > 0.99) with cell numbers estimated from additional curves. The curves prepared on the basis of DNA from one strain only led to a slight overestimation of cell numbers. The ratios of cell numbers estimated from the ‘P’ or ‘M’ curves to cell numbers estimated from the MP curves were 1.16 for PC-IGS, 1.13 for *mcy*B, 1.41 for 16S rRNA, and 1.53 for *mcy*A. In contrast, the curves prepared with the addition of the environmental background factor led to the underestimation of cell numbers. The ratios of cell numbers from the MP+L curve to cell numbers from the MP curve were 0.43 for PC-IGS, 0.85 for *mcy*B, 0.83 for 16S rRNA genes, and 0.80 for *mcy*A. The effects of mixing the DNA from various taxa, and the impacts of any residual contaminations from environmental samples, were very small. On a logarithmic scale, used in the quantification by real-time PCR, such differences are negligible (Fig. S2).

In the case of PC-IGS, underestimation in the presence of the natural background factor was the largest at end of the curve, which quantifies the lowest cell numbers (e.g. Cq = 32 − MP curve 198 cells/reaction, MP+L curve 6 cells/reaction) (Fig. S2). This might be caused by the presence of small numbers of *Microcystis* in the environmental DNA. For the final quantification, we decided not to use MP+L curves. qPCR results showed that environmental samples in which *Microcystis* or *Planktothrix* were not detected by microscope may nevertheless contain a small amount of these taxa. Errors in quantification resulting from the presence of additional DNA from studied taxa in the template DNA used to create standard curves would be larger and more difficult to estimate than a slight overestimation of cell number resulting from negligence of the impact of environmental factors (in quantification with MP curves).

**Comparison of qPCR and microscopic results.** Comparison of *Microcystis* and *Planktothrix* total cell numbers in environmental samples, estimated by microscopic observations and qPCR experiments, showed a statistically significant correlation for both studied taxa (Fig. S3). However, in the case of *Microcystis*, qPCR allowed the detection of small numbers of cells in the samples, in which the presence of this taxon was not observed microscopically. Higher sensitivity of qPCR could have resulted from the fact that for DNA isolation we used a much larger lake water volume (250 mL) than for the microscopic analysis (5−20 mL), which allowed us to detect taxa occurring in low densities. Additionally, *Microcystis* in the natural environment lives in large colonies, which may lead to a reduction in accuracy of counting low-volume samples. In contrast, the *Microcystis* strain used to create standard curves does not form colonies in laboratory conditions, so that microscopic counting was not subject to error, which may have occurred in the case of environmental samples.

In the case of *Planktothrix,* we obtained a higher correlation coefficient between the number of cells in microscopy and in qPCR, than in the case of *Microcystis* (Fig. S3b). The correlation coefficient was 0.68, and after subtracting one sample, which was most likely incorrectly microscopically counted (Mikołajskie, September 2013), the correlation coefficient was as high as 0.97. Unlike those of *Microcystis*, microscopically estimated *Planktothrix* numbers were on average two times higher than in molecular analyses. Also, other authors^24,27^ have reported higher numbers of cyanobacterial cells from microscopic counts than from qPCR.

**Statistical analyses.** The Student’s *t*-test, Spearman’s rank-order correlations and principal component analysis (PCA) were performed to determine whether there were any statistical relationships between the number of *Planktothrix* and *Microcystis* cells, cells bearing *mcy* genes, share of cells with *mcy* genes, as well as MC concentrations and environmental variables. In Spearman’s rank-order correlations, we analyzed values of the studied parameters for each sampling site and date. The parameters were: TSI, chlorophyll *a*, air and water temperature, the time of sampling ‒ meaning the phase of the vegetation season (time), Secchi depth (SD), TP, TKN, P-PO_4_, NH_4_^+^, NO_3_^-^, microcystin concentrations by ELISA (MC) in the water and total MC, total cyanobacterial cell numbers, *Planktothrix* and *Microcystis* cell numbers from the microscope analyses and from qPCR analyses (16S rRNA gene for *Planktothrix,* and PC-IGS for *Microcystis*), *Planktothrix* and *Microcystis* cells bearing toxicity genes, *mcy*A for *Planktothrix* and *mcy*B for *Microcystis*, percentage of cells with *mcy*-genes in both genera and within the total cyanobacterial cell numbers. The results were statistically significant at *P* < 0.05 (Table S9). In PCA, we analyzed most of the environmental data and data for potentially toxic genera from the qPCR analyses from three years study, but without MC, NH_4_^+^, or NO_3_^-^ results, which were for one or two years of the study, or the results from microscopic analyses. For the statistical analyses, we used the Statistica 10.8 software package.

| 2011–2013  Lake | Area  (ha) | Max depth  (m) | Mean depth  (m) | TP  Spring  (µg L^-1^) | TP Summer  (µg L^-1^) | chl *a*  Spring  (µg L^-1^) | chl *a*  Summer  (µg L^-1^) | SD  Spring  (m) | SD  Summer  (m) | Phyto  Biom  Spring (mg L^-1^) | Phyto  Biom  Summer  (mg L^-1^) | Cy share  Spring  (%) | Cy share  Summer  (%) | TSI  Spring | TSI  Summer |
| --- | --- | --- | --- | --- | --- | --- | --- | --- | --- | --- | --- | --- | --- | --- | --- |
| Mamry | 2,504 | 44 | 12 | 18±5 | 21±9 | 11±9 | 9±5 | 2.3±1.5 | 2.6±0.8 | 0.9±0.2 | 1.4±0.7 | 23±30 | 44±42 | 47±4 | 48±5 |
| Kisajno | 1,896 | 25 | 8 | 26±7 | 25±5 | 7±4 | 8±4 | 2.4±1.6 | 2.8±0.4 | 2.2±1.9 | 2.6±1.1 | 34±45 | 43±37 | 48±4 | 49±2 |
| Niegocin | 2,600 | 40 | 10 | 34±16 | 41±14 | 15±10 | 15±7 | 2.2±0.6 | 1.8±0.3 | 0.6±0.5 | 2.7±1.3 | 3±1 | 16±15 | 53±6 | 55±2 |
| Tałtowisko | 327 | 40 | 14 | 47±15 | 30±8 | 22±13 | 19±9 | 1.9±0.5 | 1.8±0.4 | 1.0±0.6 | 5.3±2.0 | 3±2 | 48±26 | 56±4 | 54±2 |
| Tałty | 1,160 | 45 | 14 | 54±21 | 36±8 | 43±25 | 25±8 | 1.7±0.2 | 1.5±0.3 | 3.9±4.4 | 5.7±2.7 | 16±20 | 37±24 | 59±5 | 57±1 |
| Mikołajskie | 498 | 27 | 11 | 58±31 | 38±14 | 40±19 | 26±9 | 1.5±0.1 | 1.5±0.4 | 3.7±1.0 | 5.3±3.7 | 26±27 | 38±31 | 60±4 | 57±3 |
| Bełdany | 941 | 46 | 10 | 55±11 | 38±9 | 29±12 | 28±6 | 1.6±0.2 | 1.7±0.4 | 2.3±1.2 | 5.0±1.9 | 36±33 | 42±34 | 59±1 | 57±2 |
| Śniardwy | 11,340 | 24 | 6 | 26±4 | 40±6 | 16±7 | 25±11 | 1.9±0.1 | 1.8±0.4 | 4.6±4.6 | 4.0±0.9 | 70±22 | 57±24 | 53±2 | 57±3 |

**Table S1.** **Basic morphological parameters, mean spring and summer values for chlorophyll *a* (chl a), total phosphorus (TP) and Secchi depth (SD), as well as mean trophic state index (TSI) and biomass of phytoplankton (Phyto biom), share of cyanobacteria in phytoplankton (% Cy share) of the studied lakes.** Presented are means from 2011, 2012 and 2013 with STDV.

| Year | Month | Mamry | Kisajno | Niegocin | Ryńskie | Tałtowisko | Tałty | Mikołajskie | Bełdany | Śniardwy |
| --- | --- | --- | --- | --- | --- | --- | --- | --- | --- | --- |
| 2011 | V | - | + | + | n.a. | - | - | - | - | + |
|  | VII | + | + | + | + | + | + | + | + | + |
|  | VIII | + | + | + | + | + | + | + | + | + |
|  | IX | + | + | + | n.a. | + | + | + | + | + |
| 2012 | IV | n.a. | n.a. | + | n.a. | + | + | + | + | + |
|  | V | + | + | + | n.a. | - | + | + | + | + |
|  | VII | + | + | + | n.a. | + | + | + | + | + |
|  | VIII | + | + | + | n.a. | + | + | + | + | + |

**Table S2. Presence of *mcy* genes (*mcy*A, *mcy*D, *mcy*E) in samples from Mazurian Lakes.** n.a. – not analyzed

| Species | Toxin | References |
| --- | --- | --- |
| *Snowella lacustris* | Microcystins | 34 |
| *Aphanocapsa sp.* | Microcystins | 33 |
| *Woronichinia naegeliana* | Microcystins | 42 |
| *Microcystis aeruginosa* | Microcystins | 32, 41 |
| *Microcystis viridis* | Microcystins | 39, 41 |
| *Microcystis wesenbergii* | Microcystins | 39 |
| *Microcystis flos-aquae* | Microcystins | 34 |
| *Microcystis ichtyoblabe* | Microcystins | 34 |
| *Microcystis smithii* | Microcystins | 36 |
| *Synechococcus sp.* | Microcystins | 30, 40 |
| *Dolichospermum flos-aquae* | Anatoxin-a, anatoxin-a(S), microcystins | 34, 41 |
| *Dolichospermum lemmermannii* | Anatoxin-a, anatoxin-a(S) | 31 |
| *Aphanizomenon flos-aquae* | Saxitoxin, cylindrospermopsin, anatoxin-a | 35 |
| *Aphanizomenon gracile* | Saxitoxin, cylindrospermopsin | 29, 41 |
| *Cuspidothrix issatschenkoi* | Saxitoxin, anatoxin-a | 38 |
| *Pseudanabaena limnetica* | Microcystins | 28, 37 |
| *Pseudanabaena catenata* | Unknown toxin | 43 |
| *Pseudanabaena mucicola* | Microcystins | 40 |
| *Planktothrix agardhii* | Microcystins, anatoxina | 34, 41 |
| *Planktothrix suspensa* | Microcystins | 44 |
| *Limnothrix redekei* | Microcystins | 45 |

**Table S3. Potentially toxic cyanobacteria taxa present in cyanobacterial community from Mazurian Lakes.** Based on several references.

| Year | Month | Mamry | | Kisajno | | Niegocin | | Ryńskie | | Tałtowisko | | Tałty | | Mikołajskie | | Bełdany | | Śniardwy | |
| --- | --- | --- | --- | --- | --- | --- | --- | --- | --- | --- | --- | --- | --- | --- | --- | --- | --- | --- | --- |
|  |  | tot | *dis* | tot | *dis* | tot | *dis* | tot | *dis* | tot | *dis* | tot | *dis* | tot | *dis* | tot | *dis* | tot | *dis* |
| 2012 | V | - | - | - | - | - | - | - | - | - | - | - | - | - | - | - | - | - | - |
|  | VI | - | - | - | - | - | - | - | - | - | - | - | - | - | - | - | - | - | - |
|  | VII | - | 0.0 | - | 0.1 | - | 0.1 | - | - | - | 0.1 | - | 0.3 | - | 0.0 | - | 0.1 | - | - |
|  | VIII | - | 0.0 | - | 0.0 | - | 0.0 | - | - | - | 0.0 | - | 0.0 | - | 0.0 | - | 0.1 | - | - |
| 2013 | V | 0.0 | 0.0 | 0.4 | 0.1 | - | 0.3 | - | - | - | 0.2 | - | 0.5 | 0.2 | 0.1 | 0.1 | 0.0 | 0.1 | 0.0 |
|  | VII | 0.0 | 0.1 | 0.2 | 0.1 | 0.1 | 0.1 | 1.9 | 0.6 | 0.7 | 0.1 | 1.5 | 0.1 | 1.5 | 0.2 | 1.5 | 0.2 | 0.1 | 0.1 |
|  | VIII | 0.0 | 0.0 | 0.1 | 0.0 | 0.8 | 0.0 | 1.7 | 0.3 | 0.6 | 0.1 | 0.7 | 0.1 | 0.4 | 0.0 | 1.0 | 0.1 | 0.1 | 0.1 |
|  | IX | 0.0 | 0.0 | 0.3 | 0.0 | 1.1 | 0.0 | - | - | 0.1 | 0.0 | 1.6 | 0.1 | 2.1 | 0.1 | 1.9 | 0.0 | 0.3 | 0.0 |

**Table S4.** **Concentrations of MCs dissolved in water and total concentrations of MCs (in cells and dissolved in water) by ELISA.** tot – analyses of MCs dissolved in water and intracellular; dis – analyses of MCs dissolved in water; in 2012, only concentration of MCs dissolved in water were analyzed; ‘-‘ – not analyzed.

| Year | Month |  | Mamry | Kisajno | Niegocin | Ryńskie | Tałtowisko | Tałty | Mikołajskie | Bełdany | Śniardwy | Year | Month | Mamry | Kisajno | Niegocin | Ryńskie | Tałtowisko | Tałty | Mikołajskie | Bełdany | Śniardwy |
| --- | --- | --- | --- | --- | --- | --- | --- | --- | --- | --- | --- | --- | --- | --- | --- | --- | --- | --- | --- | --- | --- | --- |
| 2012 | V | MC-YR  MC/LR  MC-RR  MC-VR  [Asp^3^]MC-RR  [dha^7^]MC-RR |  | X |  |  |  |  | X | X  X  X | X | 2013 | V |  | X | X |  |  |  | X | X | X |
|  | VI | MC-YR  MC/LR  MC-RR  MC-VR  [Asp^3^]MC-RR  [dha^7^]MC-RR |  |  |  |  |  |  | X  X  X |  |  |  | VII |  | X | X  X | X | X | X  X | X  X | X  X | X |
|  | VII | MC-YR  MC/LR  MC-RR  MC-VR  [Asp^3^]MC-RR  [dha^7^]MC-RR |  |  |  |  |  |  | X |  |  |  | VIII |  | X  X  X | X | X  X  X  X | X | X  X | X | X | X |
|  | VIII | MC-YR  MC/LR  MC-RR  MC-VR  [Asp^3^]MC-RR  [dha^7^]MC-RR |  | X | X  X  X |  |  |  | X | X |  |  | IX |  | X  X  X  X | X |  | X | X  X | X | X | X |

**Table S5.** **MC variants obtained by using the LC-MS/MS method**.

| Target | Locus | Forward primer/Reverse primer/TNA probe  (5'-3') | Amplicon size (bp) | Reference |
| --- | --- | --- | --- | --- |
| Total *Microcystis* | *cpc*-*IGS* | 188F  CGTACTTCGACCGCGCC  254R  TCCTACGGTTTAATTGAGACTAGCC  PCMC  LCRed610-CCGCTGCTGTCGCCTAGTCCCTG-BHQ2 | 66 | 21 |
| Potentially toxic *Microcystis* | *mcy*B | 30F  CCTACCGAGCGCTTGGG  108R  GAAAATCCCCTAAAGATTCCTGAGT  MCYBMC  FAM-CACCAAAGAAACACCCGAATCTGAGAGG-BHQ1 | 78 | 21 |
| Total *Planktothrix* | 16S rRNA | F16S  ATCCAAGTCTGCTGTTAAAGA  R16S  CTCTGCCCCTACTACACTCTAG  P16S  FAM-AAAGGCAGTGGAAACTGGAAG-BHQ1 | 82 | 22, 25 |
| Potentially toxic *Planktothrix* | *mcy*A | MAPF  CTAATGGCCGATTGGAAGAA  MAPR  CAGACTATCCCGTTCCGTTG  MAPTaq  LCCyan500-CTCTGCGGTTACAGCTAACGGGTGG-BHQ1 | 140 | 23, 25 |

**Table S6. Primers and probes used in this study for qPCR analysis.**

| Target | Efficiency (%) | Slope | y-intercept | R |
| --- | --- | --- | --- | --- |
| *cpc-IGS* | 102.8 | -3.194 | 40.29 | 0.997 |
| *mcy*B | 101.9 | -3.237 | 40.66 | 0.996 |
| 16S rRNA | 101.3 | -3.262 | 37.13 | 0.996 |
| *mcy*A | 98.9 | -3.378 | 38.48 | 0.996 |

**Table S7. Parameters of the main (MP) standard curves for qPCR assays.**

| Target | Standard curve variant | Efficiency (%) | Slope | y-intercept | R |
| --- | --- | --- | --- | --- | --- |
| PC-IGS | MP  M  MP+L | 102.8  96.0  96.5 | -3.194  -3.533  -3.501 | 40.29  40.69  35.06 | 0.997  0.998  0.997 |
| *mcy*B | MP  M  MP+L | 101.9  95.6  102.5 | -3.237  -3.555  -3.201 | 40.66  41.29  42.39 | 0.996  0.998  0.973 |
| 16S rRNA | MP  P  MP+L | 101.3  93.0  99.0 | -3.262  -3.710  -3.373 | 37.13  39.45  34.70 | 0.996  0.998  1.000 |
| *mcy*A | MP  P  MP+L | 98.9  92.4  97.1 | -3.378  -3.753  -3.469 | 38.48  41.56  36.13 | 0.996  0.997  1.000 |

**Table S8**. **Parameters of main (MP) and additional (M or P, MP+L) standard curves for qPCR assays.** MP – standard curve made from mixed DNA from *Microcystis* and *Planktothrix* strains, M – standard curve made from DNA from *Microcystis* strain, P – standard curve made from DNA from *Planktothrix* strain, MP+L – standard curve made from mixed DNA from *Microcystis* and *Planktothrix* strains with addition of environmental DNA without *Microcystis* and *Planktothrix.*

| Variable | Cyanobacteriae | *Microcystis* | *Planktothrix* | *Plantkothrix + Microcystis* | *Microcystis*-PC-IGS | *Microcystis*-*mcy*B | % of tox. *Microcystis* | *Planktothrix*-16S | *Planktothrix*-*mcy*A | % of tox. *Planktothrix* | % of M*icrocystis* in total cyanobacteria | % of tox. *Microcystis* in total cyanobacteria | % of *Planktothrix* in total cyanobacteria | % of tox. *Planktothrix* in total cyanobacteria |
| --- | --- | --- | --- | --- | --- | --- | --- | --- | --- | --- | --- | --- | --- | --- |
| MC water | **-0.52** | 0.07 | 0.23 | 0.22 | 0.06 | -0.14 | -0.27 | 0.30 | 0.30 | 0.03 | 0.19 | -0.05 | **0.47** | **0.52** |
| MC total | 0.32 | 0.18 | 0.44 | 0.40 | -0.21 | -0.40 | -0.41 | **0.87** | **0.88** | -0.07 | -0.11 | -0.40 | **0.76** | **0.75** |
| TSI | 0.23 | -0.14 | 0.26 | 0.24 | -0.10 | -0.25 | -0.27 | **0.39** | **0.40** | 0.08 | -0.21 | -0.32 | **0.37** | **0.37** |
| Time | 0.24 | 0.15 | -0.01 | -0.02 | 0.34 | 0.15 | 0.14 | 0.27 | 0.24 | **-0.62** | 0.25 | 0.23 | 0.07 | 0.02 |
| Temp water | 0.18 | 0.16 | **0.37** | **0.37** | **0.39** | 0.17 | 0.06 | 0.32 | 0.30 | **-0.35** | 0.23 | 0.17 | 0.14 | 0.14 |
| Temp air | 0.09 | 0.09 | 0.33 | 0.34 | 0.23 | 0.14 | 0.10 | 0.13 | 0.11 | **-0.41** | 0.19 | 0.21 | -0.03 | -0.05 |
| TP | -0.14 | -0.18 | 0.01 | -0.01 | -0.06 | -0.22 | -0.26 | 0.21 | 0.22 | 0.07 | -0.09 | -0.26 | **0.42** | **0.42** |
| PO_4_-P | -0.26 | -0.22 | -0.27 | -0.27 | -0.23 | -0.24 | -0.19 | -0.12 | -0.12 | 0.13 | -0.09 | -0.19 | 0.13 | 0.11 |
| TKN | -0.18 | 0.00 | 0.10 | 0.09 | 0.02 | -0.16 | -0.19 | 0.28 | 0.28 | -0.32 | 0.02 | -0.13 | **0.36** | 0.35 |
| SD | **-0.58** | 0.08 | **-0.45** | **-0.43** | 0.11 | 0.23 | 0.22 | **-0.58** | **-0.57** | 0.07 | **0.39** | **0.36** | -0.17 | -0.17 |
| Chl. *a* | 0.26 | -0.14 | 0.17 | 0.15 | -0.10 | -0.20 | -0.19 | **0.36** | **0.37** | 0.08 | -0.20 | -0.24 | 0.26 | 0.25 |
| NO_3_^-^ | 0.04 | -0.21 | -0.06 | -0.06 | 0.20 | 0.04 | 0.09 | -0.21 | -0.22 | -0.44 | 0.33 | 0.06 | 0.16 | 0.06 |
| NH_4_^+^ | -0.29 | 0.49 | -0.14 | -0.14 | 0.02 | 0.02 | 0.07 | -0.21 | -0.21 | **-0.86** | 0.17 | 0.22 | -0.19 | -0.24 |

**Table S9.** **Table Spearman’s rank-order correlations**. Marked in bold correlations are significant at *P* < 0.05. MC water – microcystins dissolved in water, by ELISA, MC total – microcystins in water and intracellular, by ELISA, TSI – mean trophic state index, Time – month of the sampling, Temp air – air temperature, Temp water – water temperature, TP – total phosphorous, PO_4_-P – orthophosphates, TKN – total Kjeldahl nitrogen, SD – Secchi depth, Chl. *a –* Chlorophyll *a*, NO_3_^-^  – nitrates, NH_4_^+^ – ammonium


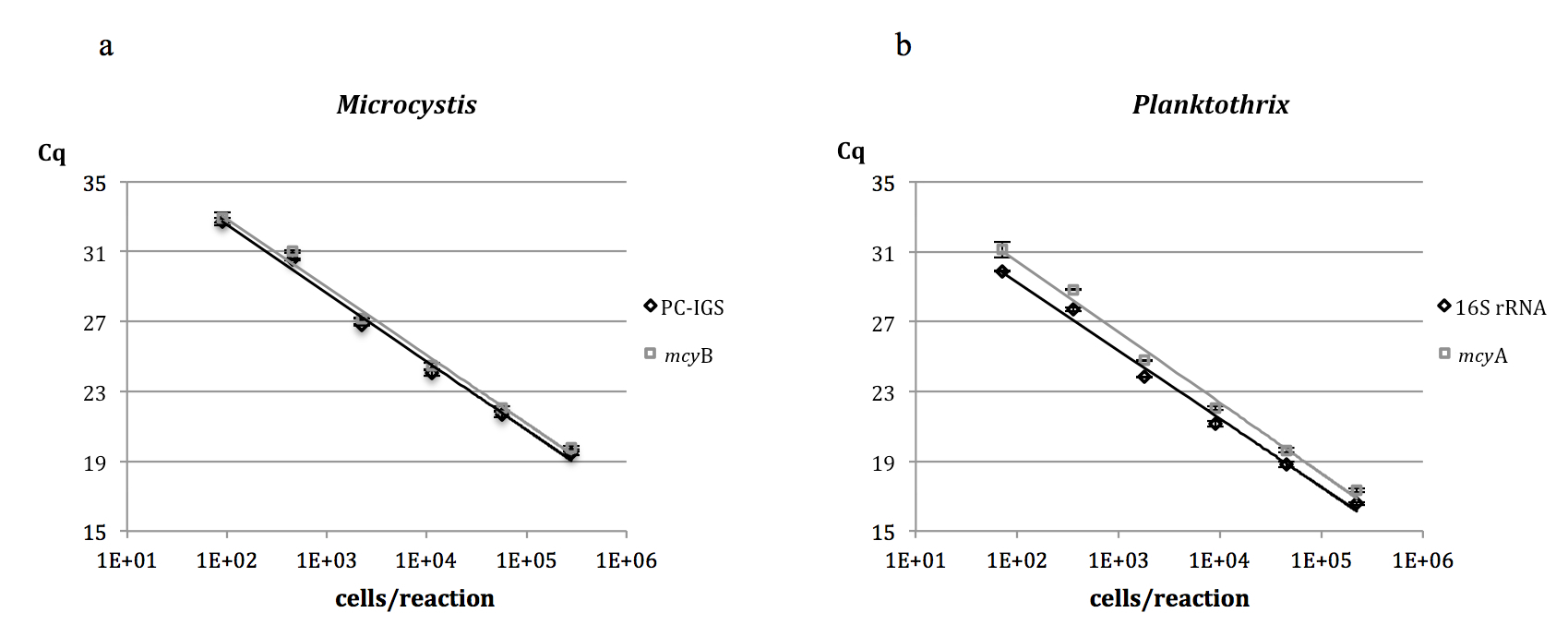


**Figure S1.** **Main (MP) standard curves for quantification.** (**a**) Total *Microcystis* (PC-IGS), potentially toxic *Microcystis* (*mcy*B); (**b**) total *Planktothrix* (16S rRNA gene) and potentially toxic *Planktothrix* (*mcy*A).

**
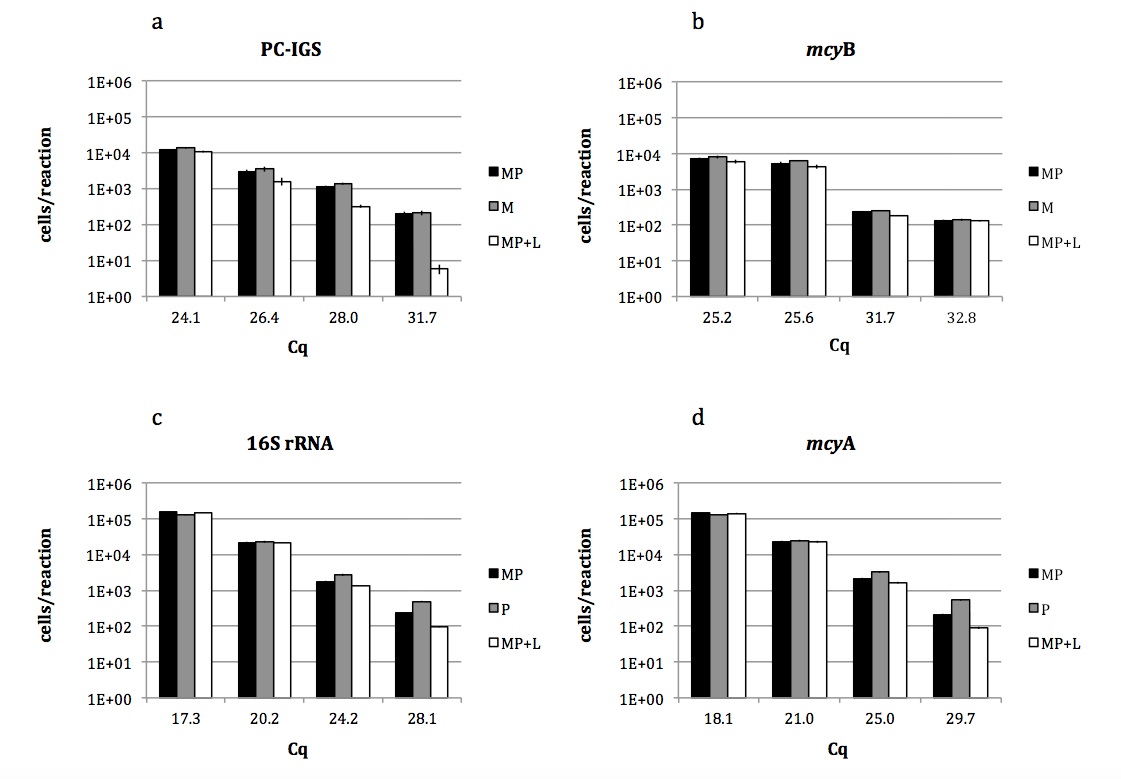
**

**Figure S2.** **Comparisons of cell numbers.** (**a**) Total *Microcystis* (PC-IGS); (**b**) potentially toxic *Microcystis* (*mcy*B); (**c**) total *Planktothrix* (16S rRNA); (**d**) potentially toxic *Planktothrix* (*mcy*A) estimated on the basis of main (MP) and additional (M, P and MP+L) standard curves.


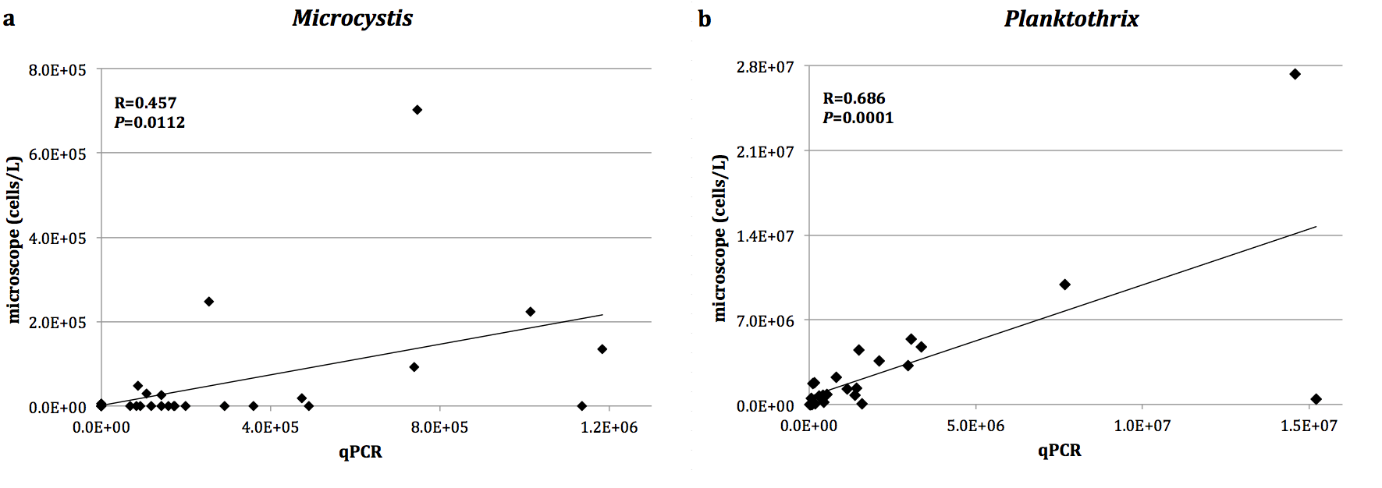


**Figure S3.** **Regression of cell numbers determined by microscope observations versus cell numbers determined by qPCR assays**. (**a**) PC-IGS for *Microcystis;* (**b**) 16S rRNA for *Planktothrix*.


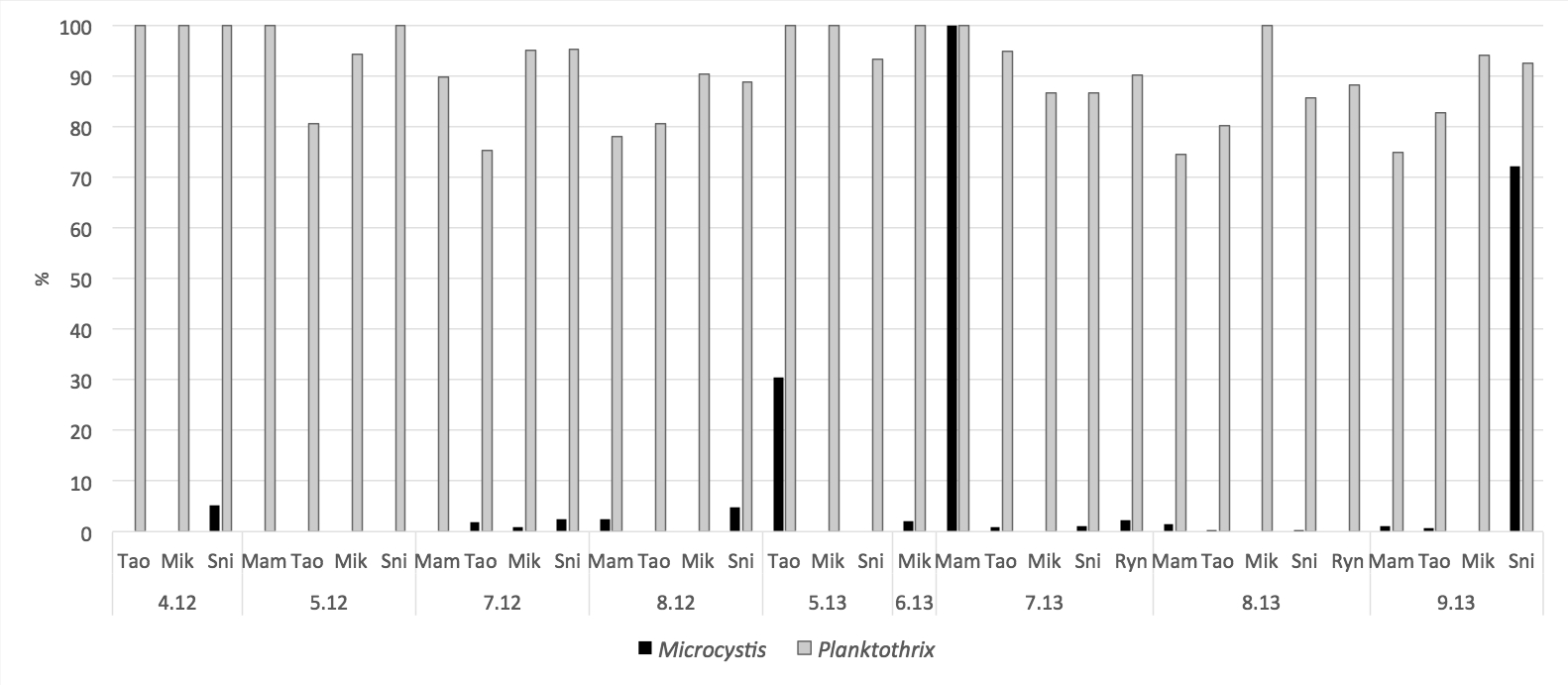


**Figure S4.** The percentage of cells with *mcy*A genes in the total number of *Planktothrix* (16S rRNA) – light grey bars, and cells with *mcy*B in the total number of *Microcystis* (PC-IGS) – black bars. Mam – Lake Mamry, Tao – Lake Tałtowisko, Mik – Lake Mikołajskie, Sni – Lake Śniardwy, Ryn – Lake Ryńskie. 4.12 means April 2012 and likewise for other dates.

**References**

1. Koroleff, F. Determination of phosphorus in *Methods in seawater analysis*, 2nd edn. (ed. Grasshoff, K., Ehrhardt, M., Kremling, K. & Almgren, T.) (Verlag Chemie International, Weinheim, 1983a).
2. Koroleff, F. Determination of ammonia in *Methods in seawater analysis*, 2nd edn. (ed. Grasshoff, K., Ehrhardt, M., Kremling, K. & Almgren, T.) (Verlag Chemie International, Weinheim, 1983b).
3. Solorzano, L. Determination of ammonia in natural waters by the phenolhypochlorite method. *Limnol Oceanogr* **14**, 799−801, doi:10.4319/lo.1969.14.5.0799 (1969).
4. Taras, M. J. Phenoldisulfonic acid method of determining nitrate in water. Photometric study. *Anal Chem* **22**, 1020−1022, doi:10.1021/ac60044a014 (1950).
5. Arar, E. J. & Collins, G. B. *Method 445.0: In vitro determination of chlorophyll a and pheophytin a in marine and freshwater phytoplankton by fluorescence* (National Exposure Research Laboratory, Office of Research and Development, U.S. Environmental Protection Agency, Cincinnati, Ohio, 1992).
6. Carlson, R. E. A trophic state index for lakes. *Limnol Oceanogr* **22**, 361−369, doi:10.4319/lo.1977.22.2.0361 (1977).
7. Utermöhl, H. Zur Vervollkommnung der quantitativen Phytoplankton-Methodik. *Mitt Int Verein Limnol* **9**, 1−38, ISSN 0538-4680 (1958).
8. Kawecka, B. & Eloranta, P. V. *Outline of the algal ecology of freshwater and land habitats* (Wydawnictwo Naukowe PWN, Warszawa, in Polish, 1994).
9. Hutorowicz, A. *Phytoplankton Metric of ecological status assessment for Polish Lakes and its performance along nutrient gradients* (Wydawnictwo Instytut Rybactwa Śródlądowego, Olsztyn, in Polish, 2005).
10. Wetzel, R. G. & Likens, G. E. *Limnological analyses* (Springer-Verlag, New York, 1991).
11. Stanier, R. Y., Kunisawa, R., Mandel, M. & Cohen-Bazire, G. Purification and properties of unicellular blue-green algae (Order Chroococcales). *Bacteriol Rev* 35. 171−205 (1971).
12. Hisbergues, M., Christiansen, G., Rouhiainen, L., Sivonen, K. & Börner, T. PCR-based identification of microcystin-producing genotypes of different cyanobacterial genera. *Arch Microbiol* **180**, 402−410. doi:10.1007/s00203-003-0605-9 (2003).
13. Rantala, A., Fewer, D. P., Hisbergues, M., Rouhiainen, L., Vaitomaa, J., Börner, T., Sivonen, K. Phylogenetic evidence for the early evolution of microcystin synthesis. *Proc Natl Acad Sci USA* 101, 568-573 (2004) doi:10.1073/pnas.0304489101.
14. Janse, I., Meima, M., Kardinaal, W. E. & Zwart, G. High-resolution differentiation of Cyanobacteria by using rRNA-internal transcribed spacer denaturing gradient gel electrophoresis. *Appl Environ Microbiol* **69**, 6634−6643 (2003).
15. Bukowska, A., Bielczyńska, A., Karnkowska, A., Chróst, R. J. & Jasser, I. Molecular (PCR-DGGE) versus morphological approach: analysis of taxonomic composition of potentially toxic cyanobacteria in freshwater lakes. *Aquat Biosyst* **10**, 2−11, doi: 10.1186/2046-9063-10-2 (2014).
16. Ye, W., Liu, X., Tan, J., Li D. & Yang, H. Diversity and dynamics of microcystin producing cyanobacteria in China’s third largest lake, Lake Taihu. *Harmful Algae* **8**, 637−644 (2009).
17. Edgar, R. C. Muscle: multiple sequence alignment with high accuracy and high throughput. *Nucleic Acids Res* **32**, 1792−7, doi:[10.1093/nar/gkh340](http://dx.doi.org/10.1093/nar/gkh340) (2004).
18. Tamura, K., Stecher, G., Peterson, D., Filipski, A. & Kumar, S. MEGA6: Molecular Evolutionary Genetics Analysis Version 6.0. *Mol Biol Evol* **30**, 2725−2729, doi:10.1093/molbev/mst197 (2013).
19. Guindon, S. & Gascuel, O. A simple, fast and accurate algorithm to estimate large phylogenies by maximum likelihood. *Syst Biol* **52**, 696−704, doi:10.1080/10635150390235520 (2003).
20. Anisimova, M. & Gascuel, O. Approximate likelihood-ratio test for branches: a fast, accurate, and powerful alternative. *Syst Biol* **55**, 539−52, doi: [10.1080/10635150600755453](http://dx.doi.org/10.1080/10635150600755453) (2006).
21. Šulčius, S. *et al.* Increased risk of exposure to microcystins in the scum of the filamentous cyanobacterium *Aphanizomenon flos-aquae* accumulated on the western shoreline of the Curonian Lagoon. *Mar Pollut Bull* **99**, 264−270, [doi:10.1016/j.marpolbul.2015.07.057](http://dx.doi.org/10.1016/j.marpolbul.2015.07.057) (2015).
22. Bustin, S. A. *et al*. The MIQE guidelines: minimum information for publication of quantitative real−time PCR experiments. *Clin Chem* **55**, 611−22, doi: 10.1373/clinchem.2008.112797 (2009).
23. Kurmayer, R. & Kutzenberger, T. Application of real-time PCR for quantification of microcystin genotypes in a population of the toxic cyanobacterium *Microcystis* sp. *Appl Environ Microbiol* **69**, 6723−6730, doi: 10.1128/AEM.69.11.6723−6730.2003 (2003).
24. Ostermaier, V. & Kurmayer, R. Distribution and abundance of nontoxic mutants of cyanobacteria in lakes of the Alps. *Microb Ecol* **58**, 323−333, doi:10.1007/s00248-009-9484-1 (2009).
25. Briand, E. *et al*. Temporal variations in the dynamics of potentially microcystin-producing strains in a bloom-forming *Planktothrix agardhii* (Cyanobacterium) population. *Appl Environ Microbiol* **74**, 3839−48, doi: 10.1128/AEM.02343-07 (2008).
26. Zhang, W., Lou, I., Ung, W. K., Kong, Y. & Mok, K. M. Application of PCR and real−time PCR for monitoring cyanobacteria, *Microcystis* spp. and *Cylindrospermopsis raciborskii* in Macau freshwater reservoir. *Front Earth Sci* **8**, 291−301, doi:10.1007/s11707-013-0409-4 (2014).
27. Savichtcheva, O. *et al*. Quantitative PCR enumeration of total/toxic *Planktothrix rubescens* and total cyanobacteria in preserved DNA isolated from lake sediments. *Appl Environ Microbiol* **77**, 8744−8753, doi:10.1128/AEM.06106-11 (2011).
28. Babica, P. Environmental and ecotoxicological aspects of cyanobacterial toxins - microcystins. (PhD dissertation, University of Masaryk, Brno, Czechy, 2006).
29. Ballot, A., Fastner, J. & Wiedner, CParalytic Shellfish Poisoning toxin-producing cyanobacterium *Aphanizomenon* gracile in northeast Germany. *Appl Environ Microbiol* 76, 1173−1180 (2010).
30. Carmichael, W. W. & Li, R. H. Cyanobacteria toxins in the Salton Sea. *Saline systems* **2**, 5 doi:10.1186/1746-1448-2-5 (2006).
31. Dow, C.S. & Swoboda, U. K. Cyanotoxins in: *The ecology of cyanobacteria: Their ecology in time and space* (ed. Whitton, B. A. & Pott, M.) 613–632 (Kluwer Academic Publishers Dordrecht, Holland, 2000).
32. Gorham, P. R. & Carmichael, W. W. Hazards of freshwater blue-green algae (cyanobacteria) in *Algae and Human Affairs* (ed. Lembi, C. A. & Waaland, J. R. 403−432 (Cambridge University Press, 1988).
33. Mowie, M. A. D., Mitrovic, S. M., Lim, R. P., Furey, A. & Yeo, D. C. J. Tropical cyanobacterial blooms: a review of prevalence, problem taxa, toxins and influencing environmental factors. *Journal of Limnology*, **74**, 205‒224. Doi: 10.4081/jlimnol.2014.1005 (2015).
34. Humpage, A. Toxin types, toxicokinetics and toxicodynamics in *Cyanobacterial Harmful Algal Blooms: State of the Science and Research Needs* (ed. Hudnell, H. K., Springer Science, Nowy Jork, USA, 2008).
35. Kurmayer, R. & Christiansen, G. The genetic basis of toxin production in cyanobacteria. *Freshw Rev* **2** :31−50, doi:10.1608/FRJ-2.1.2 (2009).
36. Liu, Y. *et al*. First report of microcystin production in *Microcystis smithii* Komárek and *Anagnostidis* (Cyanobacteria) from a water bloom in Eastern China. *J Environ Sci* **23**, 102–107 (2011).
37. Maršálek, B., Bláha, L. & Babica, P. Analyses of microcystins in the biomass of *Pseudanabaena limnetica* collected in Znojmo reservoir. *Czech Phycology* **3** ,195–197(2003)
38. Sivonen, K. & Borner, T. Bioactive compounds produced by cyanobacteria. In: *The Cyanobacteria Molecular Biology, Genomics and Evolution* (eds. Herrero, A. & Flore, E.). pp. 159–197. Caister Academic Press, Norfolk. (2008).
39. Otsuka, S. *et al*. Phylogenetic relationships between toxic and non-toxic strains of the genus Microcystis based on 16S to 23S internal transcribed spacer sequence. *FEMS Microbiological Letters* **172**, 15–21 (1999).
40. Oudra, B, *et al*. Detection and quantification of microcystins from cyanobacteria strains isolated from reservoirs and ponds in Morocco. *Environmental Toxicology* **17**, 32–39 (2002).
41. Rücker, J. *et al.* Concentrations of particulate and dissolved cylindrospermopsin in 21 *Aphanizomenon*-dominated temperate lakes. *Toxicon* **50**, 800–809 (2007).
42. Santos, M. C. R. , Muelle, H. & Pacheco, D. M. D. Cyanobacteria and microcystins in lake Furnas (S. Miguel island-Azores). *Limnetica* **3**, 107–118 (2012).
43. Gorham, P. R. & Carmichael, W. W. Hazards of freshwater blue-green algae. In: *Algae and Human Affairs* (ed. Lembic, C. A. & Waaaland, J. R.) p. 416. Cambridge University Press (1990).
44. Davis, T. W., Bullerjahn, G. S., Tuttle, T., McKay, R. M. & Watson, S. B. Phytoplankton community growth and toxity during *Planktothrix* blooms in Sandusky Bay, Lake Erie. *Environmental Science & Technology*, **49**. 7197‒7207. Doi: 10.1021/acs.est.5b00799.
45. Furtado, A. L. F. F., Calijuri, M. D., Lorenzi, A. S., Honda, R. Y., Genuario, D. B. & Fiore, M. F.: Morphological and molecular characterization of cyanobacteria from a Brazilian facultative wastewater stabilization pond and evaluation of microcystin production. *Hydrobiologia*, **627**, 195–209 (2009).
